# One-Electron Reduction Potentials: Calibration of Theoretical Protocols for Morita–Baylis–Hillman Nitroaromatic Compounds in Aprotic Media

**DOI:** 10.3390/molecules23092129

**Published:** 2018-08-24

**Authors:** Amauri Francisco da Silva, Antonio João da Silva Filho, Mário L. A. A. Vasconcellos, Otávio Luís de Santana

**Affiliations:** Chemistry Department, Federal University of Paraiba, João Pessoa 58051-900, Brazil; amauriquimica@gmail.com (A.F.d.S.); antonio.sjf@gmail.com (A.J.d.S.F.); mlaav@quimica.ufpb.br (M.L.A.A.V.)

**Keywords:** Morita–Baylis–Hilman Adducts, biological activity, reduction potentials, medicinal chemistry

## Abstract

Nitroaromatic compounds—adducts of Morita–Baylis–Hillman (MBHA) reaction—have been applied in the treatment of malaria, leishmaniasis, and Chagas disease. The biological activity of these compounds is directly related to chemical reactivity in the environment, chemical structure of the compound, and reduction of the nitro group. Because of the last aspect, electrochemical methods are used to simulate the pharmacological activity of nitroaromatic compounds. In particular, previous studies have shown a correlation between the one-electron reduction potentials in aprotic medium (estimated by cyclic voltammetry) and antileishmanial activities (measured by the IC_50_) for a series of twelve MBHA. In the present work, two different computational protocols were calibrated to simulate the reduction potentials for this series of molecules with the aim of supporting the molecular modeling of new pharmacological compounds from the prediction of their reduction potentials. The results showed that it was possible to predict the experimental reduction potential for the calibration set with mean absolute errors of less than 25 mV (about 0.6 kcal·mol^−1^).

## 1. Introduction

Nitro compounds have been used against various diseases for over five decades [1,2,3,4,5] due to their wide range of pharmacological activity against bacteria [6,7], fungus [8], and tumors [9,10] as well as malaria, leishmaniasis, and Chagas disease [11,12,13,14]. The pharmacological mechanism of these compounds generally involves enzymatic bioreduction facilitated by the electron acceptor strength of the nitro group, leading to formation of free radicals with preferential toxicity for invading microorganisms [15,16,17,18,19]. This mechanism of oxidative stress, which is responsible for damage to biological membranes, proteins, and DNA molecules [20,21], is the main route of pharmacologic action of this class of compounds [22,23]. An indicative of this mechanism is the experimental observation of the relationship between the reduction potential and the pharmacological action of some nitro compounds (greater biological activity correlated with the least negative potential), which can be used for modeling new bioactive compounds [24]. For this reason, electrochemical methods are considered useful tools for simulation of metabolic processes in vivo [25,26].

**Figure 1:**
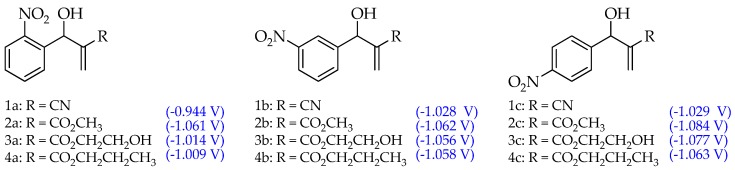
Chemical structures of the studied nitroaromatic Morita–Baylis–Hillman adducts (MBHA) and corresponding *E*_cp1_ values.

Biological antileishmaniasis activities (measured by the IC_50_) for a series of 12 nitroaromatic compounds (Figure 1)—adducts of Morita–Baylis–Hillman (MBHA) reaction—have been previously reported by researchers [25,26,27]. For this series, electrochemical studies were performed in aprotic media (*N*,*N*-dimethylformamide plus tetrabutylammonium perchlorate 0.1 mol·L^−1^ for supporting electrolyte) using cyclic voltammetry with a conventional three-electrode cell and Ag|AgCl,Cl^−^ (0.1 mol·L^−1^) as reference electrode at 25 °C (±2 °C). In these conditions, three to four reduction waves were observed. The first cathodic peak (*E*_cp1_, values in parentheses in Figure 1) was related to the generation of stable nitro-anion radicals in a diffusion-controlled reversible system. A strong relationship between antileishmaniasis IC_50_ and cathodic peak *E*_cp1_ values was observed when comparing the inter-series of MBHA compounds [25,26].

This work aims to establish protocols with low computational cost for routine prediction of one-electron reduction potentials *E*^0^_ref_ (which is related to *E*_cp1_ values) in aprotic media to assist the molecular modeling of new compounds with desired pharmacological activity.

## 2. Theoretical Fundamentals

### 2.1. Determination of Reduction Potentials from Cyclic Voltammetry

Diverse electrochemical techniques can be used for determining the standard reduction potentials (*E*^0^_ref_, where subscript “ref” is relative to the reference electrode used and superscript indicates that the species are in their standard states—concentration of 1 mol·L^−1^ in the condensed phase and temperature of 298 K) [28,29,30]. Voltammetric techniques are often employed in the case of reversible redox reactions. In a reversible cyclic voltammogram, the cathodic (*E*_cp_) and anodic (*E*_ap_) peaks relate to the standard reduction potential by the following equation:

*E*^0^_ref_ ≈ ½ (*E*_cp_ + *E*_ap_) (1)

For a redox reaction involving one electron (in standard conditions), the equation is as follows:

|*E*_cp_ − *E*_ap_| ≈ 0.059 V (2)

In this case, it is possible to relate *E*^0^_ref_ with *E*_cp_ or *E*_ap_:

*E*^0^_ref_ ≈ *E*_cp_ + 0.030 V (3)

For reversible processes, the error of this method is usually as low as 0.01–0.02 V [29].

### 2.2. Theoretical Determination of Reduction Potentials

**Scheme 1:**
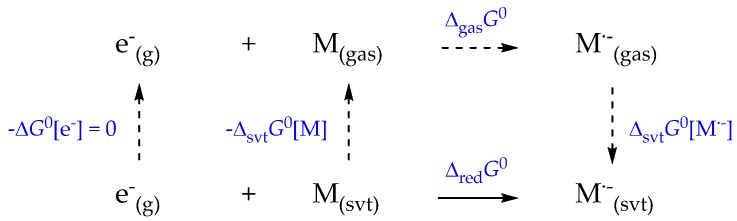
Thermodynamic cycle used to describe the composition of Δ_red_*G*^0^ and corresponding reduction potential. The solid arrow indicates the direct calculation, and dashed arrows indicate the indirect calculation path.

The computational procedures employed for predicting reduction potentials can be classified in two types [30,31,32,33,34,35,36,37,38,39]: (i) “direct”, when the reduction potential of the half-reaction for a molecular entity M is calculated directly between their solvated oxidized (M_(svt)_) and reduced (M^−^_(svt)_) forms and (ii) “indirect”, when the calculation is based on a thermodynamic cycle (Scheme 1) [40]. The direct procedure is computationally simpler but although formally correct [41], it generally leads to unsatisfactory results. For this reason, the indirect procedure is commonly employed combining different methods of electronic structure at various steps of thermodynamic cycle [42,43,44,45,46,47]. This aspect, while usually enabling a higher accuracy, also confers more complexity to the theoretical treatment and generally requires the use of computational methods of high demand for species in gas phase, making it impractical as a routine method in some cases.

The thermodynamic cycle describes the half-reaction of interest (horizontal solid arrow in Scheme 1) that occurs in solution as a result of two fundamentally different processes: reduction (horizontal dashed arrow) and solvation (vertical dashed arrows). This observation allows the deficiency of the direct procedure to be assigned to a lack of calibration of used method, especially as the basis set functions. For example, extensive basis with diffuse and polarization functions correctly describes the anionic structures in a reduction process [48,49,50], but the description of the solvation process can be affected when implicit models are used [51,52]. For this reason, a better application of the direct procedure can be obtained from a systematic study to determine the appropriate combination of methods (basis set, electronic structure, and solvation methods). Calibration procedures can also be an important step for indirect methodologies [53].

#### 2.2.1. Direct Method

The prediction of experimental standard potential—expressed by Equation (3)—under direct procedure consists of the calculation of free energy Δ_red_*G*^0^ of redox half-reaction (written by convention as a reduction reaction) for the solvated species (horizontal solid arrow in Scheme 1) [40]. A neutral compound M in a process of one electron can be expressed as follows:

M_(svt)_ + e^−^_(g)_ → M^−^_(svt)_ (4)

The absolute standard reduction potential *E*^0^_abs_ is calculated from the free energy of the reaction according to the thermodynamic relation:

*E*^0^_abs_ = −Δ_red_*G*^0^/*F* (5)

where *F* is the Faraday constant.

Experimental reduction potentials are obtained with respect to a reference, such as the normal hydrogen electrode (NHE):

H+_(aq)_ + e^−^_(g)_ → ½ H_2(g)_ (6)

with absolute reduction potential *E*^0^_NHE_ defined according to Equation (5). Experimental and theoretical estimates for this quantity still have large uncertainties (ranging from +4.80 V to +4.28 V) [28,54,55,56,57,58,59]. The most recent estimate, which is used in this study, corresponds to *E*^0^_NHE_ ≈ +4.280 V (under standard conditions). In relation to this reference electrode, the reduction potential of the species M can be expressed as follows:

*E*^0^_ref(NHE)_ = *E*^0^_abs_ − *E*^0^_NHE_ ≈ −Δ_red_*G*^0^/*F* − 4.280 V (7)

Reduction potentials of other reference electrodes are tabulated relative to the NHE. For the silver/silver chloride electrode (SSC, Ag|AgCl,Cl^−^ 0.1 mol·L^−1^) [60]:

AgCl_(s)_ + e^−^_(g)_ → Ag_(s_) + Cl^−^_(aq)_ (8)

for which,

*E*^0^_SSC(NHE)_ = *E*^0^_SSC_ − *E*^0^_NHE_ ≈ +0.222 V (9)

so that

*E*^0^_ref(SSC)_ = *E*^0^_abs_ − *E*^0^_SSC_ ≈ −Δ_red_*G*^0^/*F* − 4.502 V (10)

According to Equation (10), only the free energy of the half-reaction of Equation (4) in the direct procedure is needed to be determined computationally.

#### 2.2.2. Indirect Method

In the indirect procedure, the free energy of the half-reaction in solution (Δ_red_*G*^0^)—expressed by Equation (4)—is partitioned in three contributions: free energy of the reduction half-reaction in gas phase (Δ_gas_*G*^0^) and the solvation free energy of compound M in the neutral (Δ_svt_*G*^0^[M]) and reduced (Δ_svt_*G*^0^[M^−^]) states:

Δ_red_*G*^0^ = Δ_gas_*G*^0^ + Δ_svt_*G*^0^[M^−^] − Δ_svt_*G*^0^[M] = Δ_gas_*G*^0^ + ΔΔ_svt_*G*^0^ (11)

so that *E*^0^_ref(SSC)_ can be calculated from Equation (11) into Equation (10). This procedure is useful when different electronic structure methods are used to calculate the gas phase (with accurate level of theory) and solvation contributions (with electronic structure method and basis sets consistent with those used in the calibration of the solvation model) [42,43,44,45,46,47]. In the general case, the solvation contribution—considered the major source of inaccuracies in redox potentials predictions—should take into account the compression work necessary to bring the system from the standard state in the gas phase (under 1 atm) to the standard state in the condensed phase (under 1 mol·L^−1^) [56]. However, when the number of fragments of reactants and products that move from the gas phase to solution are the same—as in the present case—the compression terms cancels out.

## 3. Computational Details

All quantum chemistry calculations for a series of 12 MBHA with direct protocol were performed using the *Gaussian 09* [61] software. Global minima for neutral species were determined at HF/6-31+G(d) level [25]. All structures in their neutral and reduced states were reoptimized with default convergence criteria at HF [62] level, and the effect of basis set size were tested with double and triple-*zeta* Pople’s basis adding progressively diffuse and polarization functions [63]. The solvent effect was taken into account using the C-PCM [64] and SMD [65] continuum solvation models for *N*,*N*-dimethylformamide (DMF), with cavitation generated by atomic radii of UFF topological model and van der Waals surface. Frequency calculations were carried out for neutral and reduced species and used to characterize the optimized structures as stationary minima. Free energies of neutral and reduced states were obtained at 1 atm and 25 °C from the ideal gas partition functions for the structures optimized in solution [41]. The standard reduction potentials were predicted from free energies of half-reaction according to Equation (10), and the quality of the protocol was assessed by comparison theoretical with experimental reduction potentials *E*^0^_ref(SSC)_ values—estimated from cathodic peaks *E*_cp1_ and Equation (3)—considering the mean absolute errors (*e*_abs_) and standard deviation of absolute errors (*σ*_abs_). The basis sets and solvation model, which provide the smallest mean absolute errors, were selected for DFT calculations, with BP86 [66], B98 [42], B3LYP [53], PBE1PBE [67], and M06-2X [68] functionals. Dispersion effects were taken into account from Grimme’s empirical corrections of type 3 (D3, from *Gaussian* overlay 3/124) [69,70].

The gas phase contributions for indirect protocol was calculated with functionals selected from direct protocol calibration. All structures in their neutral and reduced states were reoptimized with default convergence criteria with 6-311+G(d,p) basis, and frequency calculations were carried out in order to characterize the structures as stationary minima. Corrections in the electronic energy were obtained from single point calculations with 6-311++G(3df,2pd) basis set. The solvation contributions was determined from HF and DFT calculations (with the same functional used for gas phase contribution) with two basis sets: 6-31G(p) (the same employed in the continuum solvation model calibration) [5,6] and 6-31+G(p) (to take into account the anionic products). The solvent effect was taken into account using the continuum solvation model selected from direct protocol calibration, with the same cavitation parameters. All structures in their neutral and reduced states were reoptimized with default convergence criteria for each method used, followed by frequency calculations to characterize the structures as stationary minima. Free energies of neutral and reduced states were obtained at 1 atm and 25 °C from the ideal gas partition functions, and the standard reduction potentials were predicted from free energies of half-reaction according to Equations (10) and (11). The quality of the protocol was assessed by comparison theoretical with experimental reduction potentials values considering the mean absolute errors (*e*_abs_) and standard deviation of absolute errors (*σ*_abs_).

## 4. Results

The mean absolute errors and standard deviation of absolute errors—obtained at HF level with C-PCM and SMD solvation models—are summarized in Table 1 (the complete data set are shown in Supporting Information). With C-PCM solvation model, starting from 6-31G (BS1) basis set, a mean absolute error of about 430 mV was obtained (this error was greater than window of experimental values of 140 mV). The inclusion of a set of diffuse functions on heavy atoms (BS2) led to an increase in absolute errors by 450 mV, while the inclusion of a set of polarization functions on heavy atoms (BS3) led to an increase of approximately 70 mV. However, the combined use of polarization and diffuse functions—corresponding to 6-31+G(d) (BS4) basis set—led to a reduction in error by approximately 400 mV. The remaining mean absolute error (38 mV, ~0.88 kcal·mol^−1^) was much lower than the uncertainty in the absolute potential of hydrogen reference electrode, and the standard deviation of absolute errors (23 mV, ~0.53 kcal·mol^−1^) was comparable to experimental uncertainty of voltammetric techniques for reduction potentials determination. The basis sets 6-31+G(d,p) (BS6) and 6-31++G(d,p) (BS7) with C-PCM solvation model led to similar results. All results with SMD solvation model and direct protocol were greater than 50 mV.

The window of experimental reduction potentials investigated was quite narrow (~140 mV). In this region, an important qualitative result to be analyzed was the ordering of the theoretical potentials. Despite the fact that in some cases the differences in the reduction potentials values between different types of nitroaromatics (1–4) and isomers (*a*–*c*) were less than 20 mV (~0.5 kcal·mol^−1^), the experimental potentials followed the ordering: 1 > 4 > 3 > 2 (between groups) and *a* > *b* > *c* (between isomers). Although the results obtained with 6-31+G(d) basis set led to a relatively small mean absolute error, it provided one incorrect ordering inside the groups (1*c* > 1*b*, as shown in Table 2). The inclusion of a set of diffuse functions to light atoms increased the mean absolute error, while the inclusion of polarization functions left the error almost unchanged but with the correct ordering of potentials between isomers in all cases. The combined effect of these two additional sets kept the average error near the values obtained with 6-31+G(d) basis set but with one incorrect ordering (2*b* > 2*a*). The others tested expansions in basis sets progressively increased the average absolute errors for values greater than 50 mV.

The three basis sets that led mean absolute errors less than 50 mV were selected to perform DFT calculations. The results are summarized in Table 3 (the complete set of data are shown in Supporting Information). In general, the inclusion of empirical dispersion correction marginally reduced the mean absolute error, with the exception of B3LYP and M06-2X functionals. The B98+D3, mPW1PW91+D3 and M06-2X functionals provided better statistical results with all basis sets (with *e*_abs_ < 25 mV and *σ*_abs_ < 20 mV or ~0.6 kcal·mol^−1^ and ~0.5 kcal·mol^−1^, respectively). The results from B98+D3 functional provided one incorrect ordering inside the groups (1*b* < 1*c*) and from mPW1PW91+D3 functional two incorrect ordering (1*b* < 1*c* and 2*b* < 2*c*) with all basis set. Functional M06-2X functional provided an incorrect ordering only with 6-31+G(d) basis set. For this reason, two direct protocols were selected with the aim of achieving the lowest computational cost with highest predictive power: B98+D3/6-31+G(d) and M06-2X/6-31+G(d,p).

The two functionals that led to the lowest mean absolute errors were selected for calibration of indirect protocol. The results are summarized in Table 4 (the complete set of data are shown in Supporting Information). All results obtained with solvation contribution estimated from HF calculations presented errors greater than those obtained with the corresponding functionals. In addition, the results obtained with the basis set 6-31+G(d) resulted in the smallest mean absolute errors. This result was expected due to the presence of anionic products, which require the inclusion of diffuse functions for better description. However, the B98+D3 functional provided less satisfactory results with indirect protocol than those obtained with direct calculations (with *e*_abs_ > 50 mV and *σ*_abs_ > 30 mV).

The predicted reduction potentials at DFT level from direct and indirect calibrated protocols for investigated nitroaromatic MBHA are shown in Table 5. With B98 functional, the results obtained by direct procedure had thermochemical precision (*e*_abs_ < 43 mV or 1 kcal·mol^−1^), which did not occur with the indirect protocol. On the other hand, although the predictions obtained with the M06-2X functional had the necessary precision with both protocols, it is important to consider the greater simplicity of the direct calculation, which is consequently reflected in the considerably lower computational cost. This result is consistent with that observed in other investigations [71,72,73]. In addition, only the direct protocol with the M06-2X functional predicted the correct ordering throughout the series.

## 5. Conclusions

In this work, the calibration of two computational protocols to predict the one-electron reduction potentials in an aprotic medium was performed from a calibration set consisting of 12 molecules that exhibit antileishmaniasis activity. The first calibrated computational protocol was based on a direct calculation procedure in which the free energies of the oxidized and reduced forms of the molecule under study were computed directly in the solvated phase. The second protocol—the most commonly used in this research area—was based on a thermodynamic cycle that makes it possible to separate the free energy of the reduction reaction in contributions from gas and solvated phases, which are computed separately at different calculation levels, assuming that an errors cancellation can produce better results. In both cases, the calibration was based on the selection of the electronic structure method, basis set, and continuous solvation model that minimizes the mean absolute error and standard deviation. The calibration set was composed of 12 compounds of interest for which the reduction potentials were experimentally determined by cyclic voltammetry covering a window of potentials of only 140 mV (approximately 3 kcal·mol^−1^ of difference between data set extremes). The results showed that the experimental reduction potential can be predicted with thermochemical accuracy (<43 mV, ~1 kcal·mol^−1^), with mean absolute errors lower than 25 mV (lower than uncertainty in the absolute potential of reference hydrogen electrode, without inclusion of corrective terms) and standard deviation of the order of 15 mV (comparable to experimental uncertainty).

Although several parameters are related to the biological activity of a given class of compounds, there are theoretical and experimental reasons to assume that the reduction potential is an important parameter in the pharmacological mechanism of nitroaromatic compounds. Therefore, the development of a theoretical protocol for the prediction of reduction potentials may be useful in the modeling of new nitroaromatic compounds with the desired biological activity. However, in order for the calibrated protocols to be routinely used for molecular modeling of new drug candidates from the prediction of their reduction potentials, it is necessary to verify if they are transferable to other molecular systems. This study is currently underway in our research group.

## Figures and Tables

**Table 1:** Mean absolute errors (*e*_abs_) and standard deviation of absolute errors (*σ*_abs_) for theoretical values of 12 investigated nitroaromatic Morita–Baylis–Hillman adducts (MBHA) calculated at Hartree–Fock (HF) level with C-PCM and SMD continuum solvation models from direct protocol. Values in millivolts.

	BS1 ^a^	BS2	BS3	BS4	BS5	BS6	BS7	BS8	BS9
	**HF/C-PCM**								
***e*_abs_**	433	887	506	**38**	199	**41**	**39**	66	204
***σ*_abs_**	79	36	62	**23**	36	**22**	**24**	29	38
	**HF/SMD**								
***e*_abs_**	65	478	869	410	186	408	400	431	581
***σ*_abs_**	78	57	93	54	57	63	51	57	55

^a^ BS = Basis Set: BS1 = 6-31G; BS2 = 6-31+G; BS3 = 6-31G(d); BS4 = 6-31+G(d); ^a^ BS5 = 6-31++G(d); BS6 = 6-31+G(d,p); BS7 = 6-31++G(d,p); ^a^ BS8 = 6-311++G(d,p); BS9 = 6-311+G(2df,p).

**Table 2:** Theoretical reduction potentials calculated at HF level with C-PCM continuum solvation model of 12 investigated nitroaromatic MBHA. Reduction potentials values are in volts. Mean absolute errors (*e*_abs_), standard deviation of absolute errors (*σ*_abs_), and range of experimental values (*E*^0^_max_–*E*^0^_min_) are in millivolts.

Est.	Theoretical: HF/C-PCM [V]			Experimental [V]	
	sBF1 ^a^	sBF2	sBF3	*E*_cp1_	*E* ^0^_ref(SSC)_
**1*a***	−0.929	−0.933	−0.900	−0.944	−0.915
**1*b***	−1.000	−1.013	−1.012	−1.028	−0.999
**1*c***	−0.993	−1.015	−1.014	−1.029	−1.000
**2*a***	−1.064	−1.056	−1.055	−1.061	−1.032
**2*b***	−1.080	−1.067	−1.050	−1.062	−1.033
**2*c***	−1.090	−1.084	−1.082	−1.084	−1.055
**3*a***	−0.939	−0.925	−0.926	−1.014	−0.985
**3*b***	−1.066	−1.074	−1.074	−1.056	−1.027
**3*c***	−1.096	−1.100	−1.104	−1.077	−1.048
**4*a***	−1.066	−1.066	−1.067	−1.009	−0.980
**4*b***	−1.069	−1.078	−1.074	−1.058	−1.029
**4*c***	−1.092	−1.096	−1.097	−1.063	−1.034
***e*_abs_**	38	41	39	*E*^0^_max_–*E*^0^_min_	140
***σ*_abs_**	23	22	24		

^a^ sBF = Selected Basis Functions: sBF1 = 6-31+G(d); sBF2 = 6-31+G(d,p); sBF3 = 6-31++G(d,p).

**Table 3:** Mean absolute errors (*e*_abs_) and standard deviation of absolute errors (*σ*_abs_) for theoretical values of 12 investigated nitroaromatic MBHA calculated at DFT level with C-PCM continuum solvation model from direct protocol. Values are in millivolts.

	DF1 ^a^	DF1^+^	DF2	DF2^+^	DF3	DF3^+^	DF4	DF4^+^	DF5	DF5^+^
	**6-31+G(d)/C-PCM**									
***e*_abs_**	55	61	23	20	31	27	31	24	24	27
***σ*_abs_**	26	24	20	16	22	21	23	16	14	17
	**6-31+G(d,p)/C-PCM**									
***e*_abs_**	57	65	23	21	30	27	26	24	23	27
***σ*_abs_**	24	24	18	13	21	19	17	14	14	19
	**6-31++G(d,p)/C-PCM**									
***e*_abs_**	59	63	22	23	28	26	26	25	24	28
***σ*_abs_**	24	25	17	15	21	18	18	14	14	22

^a^ DF = Density Functional: DF1 = B3LYP; DF1^+^ = B3LYP+D3; DF2 = B98; DF2^+^ = B98+D3; DF3 = PBE1PBE; DF3^+^ = PBE1PBE+D3; DF4 = mPW1PW91; DF4^+^ = mPW1PW91+D3; DF5 = M06-2X; DF5^+^ = M06-2X+D3.

**Table 4:** Mean absolute errors (*e*_abs_) and standard deviation of absolute errors (*σ*_abs_) for theoretical values of 12 investigated nitroaromatic MBHA calculated at DFT level with C-PCM continuum solvation model from indirect protocol. Values are in millivolts.

**Gas**	**B98+D3/6-311++G(3df,2pd)//B98+D3/6-311+G(d,p)**			
**SVT**	**HF/BF1 ^a^**	**HF/BF2**	**B98+D3/BF1**	**B98+D3/BF2**
***e*_abs_**	293	303	143	75
***σ*_abs_**	106	116	55	34
**Gas**	**M06-2X/6-311++G(3df,2pd)//M06-2X/6-311+G(d,p)**			
**SVT**	**HF/BF1**	**HF/BF2**	**M06-2X/BF1**	**M06-2X/BF2**
***e*_abs_**	248	221	69	16
***σ*_abs_**	153	84	40	16

^a^ BF = Base Functions: BF1 = 6-31G(d); BF2 = 6-31+G(d).

**Table 5:** Theoretical reduction potentials calculated at DFT level with C-PCM continuum solvation model of 12 investigated nitroaromatic MBHA. Reduction potentials values are in volts, mean absolute errors (*e*_abs_) and standard deviation of absolute errors (*σ*_abs_) are in millivolts.

Est.	B98/Direct ^a^	B98/Indirect ^b^	M06-2X/Direct ^c^	M06-2X/Indirect ^d^	Exp.
**1*a***	−0.931	−0.990	−0.922	−0.974	−0.915
**1*b***	−0.982	−1.041	−0.960	−0.982	−0.999
**1*c***	−0.965	−1.006	−1.008	−1.003	−1.000
**2*a***	−1.014	−1.087	−0.991	−1.026	−1.032
**2*b***	−1.030	−1.091	−1.010	−1.059	−1.033
**2*c***	−1.065	−1.170	−1.041	−1.054	−1.055
**3*a***	−0.984	−1.051	−0.969	−0.988	−0.985
**3*b***	−1.035	−1.098	−0.976	−1.006	−1.027
**3*c***	−1.075	−1.144	−1.025	−1.072	−1.048
**4*a***	−1.025	−1.086	−0.950	−0.995	−0.980
**4*b***	−1.041	−1.104	−1.011	−1.041	−1.029
**4*c***	−1.081	−1.166	−1.021	−1.044	−1.034
***e*_abs_**	20	75	23	16	_---_
***σ*_abs_**	16	34	14	16	_---_

^a^ B98+D3/6-31+G(d); ^b^ B98+D3/6-311++G(3df,2pd)//B98+D3/6-311+G(d,p)@B98+D3/6-31+G(d); ^c^ M06-2X/6-31+G(d,p); ^d^ M06-2X/6-311++G(3df,2pd)//M06-2X/6-311+G(d,p)@M06-2X/6-31+G(d).

## Supplementary Materials

The following are available online.
