# Supplementary material for: One-Electron Reduction Potentials: Calibration of Theoretical Protocols for Morita–Baylis–Hillman Nitroaromatic Compounds in Aprotic Media
Source: Molecules. 2018 Aug 24;23(9):2129. doi: 10.3390/molecules23092129 (PMC6225277; doi:10.3390/molecules23092129)
Supplement: Supplementary file 1 [file molecules-23-02129-s001.pdf]

molecules

MDPI

Article

One-Electron Reduction Potentials: Calibration of Theoretical Protocols for Morita–Baylis–Hillman Nitroaromatic Compounds in Aprotic Media

Amauri Francisco da Silva, Antonio João da Silva Filho, Mário L. A. A. Vasconcellos and Otávio Luís de Santana *

Chemistry Department, Federal University of Paraiba, João Pessoa, 58051-900, Brazil; amauriquimica@gmail.com (A.F.d.S.); antonio.sjf@gmail.com (A.J.d.S.F.); mlaav@quimica.ufpb.br (M.L.A.A.V.)

* Correspondence: otavio@quimica.ufpb.br; Tel.: +55-(83)-3216-7200

Received: 30 July 2018; Accepted: 11 August 2018; Published: date

Supplementary Material

Data S1: Computational data of the 12 AMBHs structures investigated at M062X/6-31+G(d,p) level with C-PCM continuum solvation model for N,N-dimethylformamide (DMF) at 1.00 atm and 25.00 °C.

Method: M062X/6-31+G(d,p)
Solvent: n,n-DiMethylFormamide [C-PCM]
Protocol: Direct

Pressure: 1,00 atm (Gas Phase)
Temperature: 25,00 °C

AMBHs

Group 1

==

Structure: 1a (Charge = 0)

Center Number	Atomic Symbol	Coordinates (Angstroms) X	Y	Z	Normal Mode	Frequencies (cm**-1)
1	6	1.353687	-0.202813	-0.012799	1	28.92
2	6	2.475362	0.505236	-0.439619	2	51.60
3	6	2.452453	1.892680	-0.425916	3	70.41
4	6	1.316722	2.550165	0.040838	4	107.41
5	6	0.215237	1.822608	0.482781	5	138.32
6	6	0.189802	0.425043	0.459666	6	153.24
7	1	3.315826	2.451544	-0.768719	7	213.70
8	1	1.285366	3.634151	0.068714	8	230.97
9	1	-0.641979	2.361571	0.869991	9	274.47
10	6	-1.075286	-0.285625	0.948624	10	310.70
11	6	-2.020630	-0.581470	-0.208263	11	329.66
12	1	-0.818929	-1.235050	1.415532	12	350.50
13	6	-2.501441	-1.792562	-0.500039	13	372.35
14	1	-3.202600	-1.943935	-1.313304	14	401.84
15	1	-2.186229	-2.656090	0.075984	15	427.87
16	8	-1.733319	0.465910	1.951035	16	447.57
17	1	-2.283900	1.159782	1.564128	17	520.90
18	7	-2.753857	1.521625	-1.553856	18	568.00
19	6	-2.438053	0.567723	-0.977026	19	609.34
20	1	3.345730	-0.040565	-0.782106	20	662.95
21	7	1.469042	-1.666345	-0.070936	21	686.95
22	8	0.442869	-2.329132	-0.086578	22	714.97

23	8	2.587112	-2.152312	-0.110226	23	721.41
					24	746.30
					25	761.96
					26	797.46
					27	815.21
					28	864.70
					29	887.40
					30	904.50
					31	973.70
					32	996.76
					33	1020.41
					34	1033.71
					35	1087.42
					36	1095.53
					37	1127.85
					38	1183.63
					39	1185.57
					40	1220.71
					41	1239.13
					42	1294.10
					43	1299.59
					44	1327.07
					45	1364.97
					46	1431.14
					47	1456.96
					48	1468.69
					49	1491.19
					50	1533.71
					51	1651.97
					52	1668.46
					53	1703.62
					54	1724.62
					55	2393.29
					56	3189.81
					57	3194.61
					58	3220.50
					59	3232.17
					60	3241.75
					61	3261.22
					62	3297.55
					63	3850.35

Sum of Electronic and Nuclear Energy: -720.666165 (Hartree)
Sum of Electronic and Thermal Free Energies: -720.536839
Standard Free Energy (1,0 M): -720.533820

Structure: 1a (Charge = -1)

Center Number	Atomic Symbol	Coordinates (Angstroms) X	Y	Z	Normal Mode	Frequencies (cm**-1)
1	6	-1.257010	0.263166	0.049778	1	33.67
2	6	-2.379783	-0.372955	-0.516301	2	51.58
3	6	-2.495936	-1.755556	-0.500299	3	63.94
4	6	-1.492138	-2.536463	0.073283	4	114.37
5	6	-0.358411	-1.909535	0.592830	5	139.69
6	6	-0.200400	-0.523551	0.585459	6	158.78
7	1	-3.379367	-2.222057	-0.925781	7	219.46
8	1	-1.577636	-3.617436	0.105323	8	227.53
9	1	0.425357	-2.527204	1.019278	9	277.65
10	6	1.140673	0.078186	0.994705	10	316.55
11	6	1.859205	0.587328	-0.252942	11	327.70
12	1	0.988522	0.929276	1.653068	12	355.76
13	6	2.083053	1.870449	-0.549865	13	371.29
14	1	2.600460	2.158748	-1.458736	14	401.74
15	1	1.715132	2.642174	0.117687	15	414.62
16	8	1.970008	-0.829228	1.699665	16	442.54
17	1	2.245091	-1.556375	1.123888	17	499.85
18	7	2.653477	-1.338135	-1.813267	18	538.53
19	6	2.306483	-0.453771	-1.148926	19	560.96
20	1	-3.155740	0.251873	-0.939859	20	607.29
21	7	-1.245916	1.664706	0.069172	21	652.04
22	8	-0.530688	2.292434	0.942392	22	679.79
23	8	-2.057966	2.308196	-0.698624	23	686.32
					24	729.50
					25	755.65
					26	759.73
					27	788.65
					28	803.39
					29	858.75
					30	882.43
					31	959.64
					32	972.59

33	1005.85
34	1007.85
35	1076.52
36	1080.05
37	1102.89
38	1148.18
39	1168.34
40	1196.86
41	1226.69
42	1271.68
43	1287.99
44	1304.15
45	1341.84
46	1365.80
47	1388.94
48	1416.94
49	1444.52
50	1496.83
51	1524.10
52	1631.27
53	1663.93
54	1722.56
55	2384.34
56	3183.85
57	3201.66
58	3206.06
59	3210.02
60	3228.07
61	3246.78
62	3290.93
63	3835.01

```
------------------------------------------------- -----------------
Sum of Electronic and Nuclear Energy:            -720.794064 (Hartree)
Sum of Electronic and Thermal Free Energies:     -720.668324
Standard Free Energy (1,0 M):                    -720.665305
===================================================================
===================================================================
```

Structure: 1b (Charge = 0)

```
------------------------------------------------- -----------------
Center Atomic         Coordinates (Angstroms)     Normal Frequencies
Number Symbol         X            Y            Z    Mode      (cm**-1)
------------------------------------------------- -----------------
```

1	6	-0.668151	0.198239	0.600013	1	22.95
2	6	-1.855728	-0.199225	-0.006804	2	36.67
3	6	-1.943728	-1.299727	-0.848675	3	48.35
4	6	-0.782887	-2.030617	-1.093150	4	83.26
5	6	0.420549	-1.650131	-0.505963	5	153.52
6	6	0.482025	-0.539242	0.342281	6	157.38
7	1	-2.892585	-1.571733	-1.294336	7	178.51
8	1	-0.820691	-2.895598	-1.745707	8	222.03
9	1	1.327019	-2.217041	-0.699504	9	280.77
10	6	1.816087	-0.129999	0.949809	10	291.40
11	6	2.687619	0.522255	-0.111081	11	309.52
12	1	1.630704	0.609403	1.741261	12	332.06
13	6	3.872251	0.055735	-0.513661	13	344.50
14	1	4.439218	0.560178	-1.287737	14	416.43
15	1	4.280233	-0.841707	-0.063494	15	435.27
16	8	2.532406	-1.233988	1.450539	16	499.43
17	1	2.037551	-1.617416	2.185944	17	522.39
18	7	1.666782	2.674305	-1.154243	18	547.48
19	6	2.135747	1.716669	-0.702038	19	570.87
20	1	-0.654016	1.063557	1.253980	20	622.92
21	7	-3.072242	0.579729	0.262552	21	664.56
22	8	-4.108012	0.231285	-0.280086	22	700.39
23	8	-2.985882	1.536251	1.015427	23	727.70
--					24	761.88
					25	764.48
					26	833.24
					27	854.09
					28	854.77
					29	932.42
					30	946.51
					31	972.25
					32	976.55
					33	1007.71
					34	1019.29
					35	1033.94
					36	1112.78
					37	1142.71
					38	1148.27
					39	1181.65
					40	1225.04
					41	1241.34
					42	1273.38

					43	1305.30
					44	1345.28
					45	1383.09
					46	1416.97
					47	1445.21
					48	1469.09
					49	1493.04
					50	1535.72
					51	1656.46
					52	1675.37
					53	1715.23
					54	1725.94
					55	2396.17
					56	3068.38
					57	3198.70
					58	3218.34
					59	3240.97
					60	3250.22
					61	3254.05
					62	3302.99
					63	3870.44

--- -----------------

Sum of Electronic and Nuclear Energy: -720.674636 (Hartree)

Sum of Electronic and Thermal Free Energies: -720.547076

Standard Free Energy (1,0 M): -720.544057

==

==

Structure: 1b (Charge = -1)

Center Number	Atomic Symbol	Coordinates (Angstroms) X	Y	Z	Normal Mode	Frequencies (cm**-1)
1	6	-0.730098	0.242866	0.593276	1	27.23
2	6	-1.937454	-0.135878	-0.030728	2	48.88
3	6	-1.943015	-1.206232	-0.945844	3	74.92
4	6	-0.756712	-1.876732	-1.224243	4	103.00
5	6	0.441718	-1.503256	-0.614332	5	146.96
6	6	0.442644	-0.439331	0.298639	6	156.58
7	1	-2.876067	-1.490153	-1.415628	7	173.38
8	1	-0.767937	-2.702406	-1.929702	8	215.67
9	1	1.366837	-2.027632	-0.836510	9	281.16
10	6	1.738722	-0.037321	0.982228	10	298.98

11	6	2.773086	0.407020	-0.035096
12	1	1.531213	0.813495	1.647470
13	6	3.956627	-0.180395	-0.230370
14	1	4.646572	0.181062	-0.984309
15	1	4.237237	-1.035558	0.373255
16	8	2.313695	-1.103381	1.712902
17	1	1.634603	-1.472167	2.291965
18	7	2.064909	2.463075	-1.462439
19	6	2.390913	1.545648	-0.834806
20	1	-0.739356	1.066621	1.298265
21	7	-3.115949	0.542430	0.265906
22	8	-4.211004	0.191559	-0.315433
23	8	-3.094491	1.503056	1.124852

11	311.64
12	332.30
13	347.76
14	383.69
15	415.99
16	472.82
17	500.36
18	537.07
19	550.47
20	589.40
21	641.32
22	657.56
23	693.85
24	708.63
25	764.18
26	795.03
27	804.04
28	856.60
29	886.33
30	906.12
31	918.10
32	980.26
33	1000.39
34	1000.76
35	1007.99
36	1094.12
37	1105.82
38	1129.90
39	1167.19
40	1217.12
41	1224.59
42	1270.52
43	1294.06
44	1332.76
45	1348.38
46	1385.38
47	1408.30
48	1428.99
49	1449.08
50	1506.21
51	1534.08
52	1641.57

53	1662.33
54	1728.80
55	2403.89
56	3055.30
57	3197.09
58	3206.11
59	3211.79
60	3229.84
61	3248.48
62	3312.05
63	3875.28

Sum of Electronic and Nuclear Energy: -720.802181 (Hartree)
Sum of Electronic and Thermal Free Energies: -720.677200
Standard Free Energy (1,0 M): -720.674181

Structure: 1c (Charge = 0)

Center Number	Atomic Symbol	Coordinates (Angstroms) X	Y	Z	Normal Mode	Frequencies (cm**-1)
1	6	-0.049394	-1.298092	-0.209368	1	6.11
2	6	-1.405261	-1.162714	-0.477718	2	43.19
3	6	-2.070341	-0.051994	0.032546	3	48.45
4	6	-1.436465	0.917361	0.799131	4	62.98
5	6	-0.079452	0.761351	1.063825	5	123.26
6	6	0.615910	-0.340122	0.561803	6	172.80
7	1	-1.993105	1.764105	1.181443	7	180.38
8	1	0.434492	1.502154	1.669070	8	261.78
9	6	2.106277	-0.477043	0.808774	9	293.26
10	6	2.869871	0.133445	-0.368633	10	305.25
11	1	2.365952	0.085481	1.715011	11	319.12
12	6	3.586553	-0.570854	-1.247902	12	358.96
13	1	4.099979	-0.088664	-2.071740	13	378.94
14	1	3.661299	-1.648163	-1.146225	14	413.08
15	8	2.409715	-1.841745	0.957175	15	439.39
16	1	3.305760	-1.932473	1.307407	16	500.36
17	7	2.639874	2.715300	-0.568883	17	532.59
18	6	2.755818	1.564737	-0.500594	18	557.69
19	1	0.497598	-2.152628	-0.593831	19	564.18
20	1	-1.940624	-1.895687	-1.068454	20	622.54

21	7	-3.503569	0.099572	-0.247294
22	8	-4.080184	1.069295	0.218196
23	8	-4.047853	-0.751885	-0.931701

21	637.49
22	699.88
23	721.72
24	766.35
25	769.48
26	840.78
27	849.26
28	854.93
29	892.17
30	898.95
31	964.64
32	994.07
33	1008.44
34	1014.51
35	1034.62
36	1130.06
37	1148.40
38	1152.67
39	1195.18
40	1207.57
41	1233.78
42	1258.77
43	1328.36
44	1347.41
45	1375.83
46	1429.87
47	1436.59
48	1469.54
49	1472.12
50	1547.73
51	1652.91
52	1688.77
53	1708.60
54	1722.14
55	2398.05
56	3075.26
57	3203.90
58	3209.47
59	3226.79
60	3252.11
61	3252.86
62	3306.69

					63	3870.49

Sum of Electronic and Nuclear Energy:	-720.674542 (Hartree)
Sum of Electronic and Thermal Free Energies:	-720.548181
Standard Free Energy (1,0 M):	-720.545162

Structure: 1c (Charge = -1)

Center Number	Atomic Symbol	Coordinates (Angstroms) X	Y	Z	Normal Mode	Frequencies (cm**-1)
1	6	0.064095	-0.830504	-0.663987	1	33.84
2	6	-1.289529	-0.706685	-0.931661	2	40.44
3	6	-2.128984	-0.058429	0.000255	3	72.44
4	6	-1.581192	0.450267	1.194466	4	110.33
5	6	-0.221681	0.305967	1.441738	5	115.02
6	6	0.618421	-0.332132	0.524173	6	150.33
7	1	-2.231894	0.946635	1.902926	7	192.48
8	1	0.193768	0.698174	2.366985	8	230.71
9	6	2.094992	-0.492573	0.803813	9	295.55
10	6	2.922333	0.147738	-0.307333	10	304.86
11	1	2.328591	0.029189	1.742092	11	317.26
12	6	3.706185	-0.515507	-1.161740	12	342.16
13	1	4.251309	-0.002222	-1.945587	13	363.93
14	1	3.800700	-1.593175	-1.082735	14	430.75
15	8	2.391952	-1.871680	0.919678	15	431.78
16	1	3.282557	-1.974271	1.282079	16	446.52
17	7	2.652567	2.729627	-0.474974	17	517.87
18	6	2.782950	1.579800	-0.417978	18	536.30
19	1	0.708528	-1.322033	-1.389487	19	558.04
20	1	-1.719622	-1.092203	-1.847241	20	565.31
21	7	-3.488198	0.080395	-0.262273	21	623.42
22	8	-4.242151	0.670727	0.599647	22	642.04
23	8	-3.971057	-0.380032	-1.364171	23	683.76
					24	737.60
					25	763.49
					26	799.18
					27	833.16
					28	840.33
					29	857.82
					30	872.43

```
                                                    31      962.34
                                                    32      980.51
                                                    33      995.27
                                                    34     1006.00
                                                    35     1016.81
                                                    36     1095.97
                                                    37     1112.91
                                                    38     1128.43
                                                    39     1191.27
                                                    40     1197.30
                                                    41     1235.61
                                                    42     1252.56
                                                    43     1310.64
                                                    44     1334.43
                                                    45     1365.03
                                                    46     1384.19
                                                    47     1415.26
                                                    48     1433.73
                                                    49     1447.90
                                                    50     1476.53
                                                    51     1555.54
                                                    52     1632.39
                                                    53     1671.63
                                                    54     1720.42
                                                    55     2393.83
                                                    56     3065.02
                                                    57     3192.04
                                                    58     3201.98
                                                    59     3206.91
                                                    60     3250.53
                                                    61     3262.44
                                                    62     3303.23
                                                    63     3857.30
------------------------------------------------- -----------------
Sum of Electronic and Nuclear Energy:             -720.801822 (Hartree)
Sum of Electronic and Thermal Free Energies:      -720.676590
Standard Free Energy (1,0 M):                     -720.673571
====================================================================
```

Group 2

```
====================================================================
```

Structure: 2a (Charge = 0)

Center Number	Atomic Symbol	Coordinates (Angstroms) X	Y	Z	Normal Mode	Frequencies (cm**-1)
1	6	1.915819	0.016113	-0.202098	1	23.09
2	6	2.772879	0.932003	-0.811131	2	38.09
3	6	2.500054	2.288279	-0.716285	3	64.63
4	6	1.389555	2.706679	0.014099	4	81.41
5	6	0.558452	1.775651	0.629648	5	115.44
6	6	0.778457	0.396629	0.530036	6	136.58
7	1	3.636576	0.567534	-1.353490	7	156.55
8	1	3.152579	3.007627	-1.198078	8	160.04
9	1	1.168689	3.764434	0.112880	9	184.50
10	1	-0.285079	2.130850	1.208199	10	218.03
11	6	-0.181657	-0.559043	1.255983	11	236.97
12	6	-1.315002	-1.026223	0.349778	12	280.75
13	1	0.369742	-1.441966	1.577673	13	291.09
14	6	-1.503997	-2.302869	0.010021	14	348.08
15	1	-2.345832	-2.597697	-0.607156	15	367.51
16	1	-0.819752	-3.074528	0.345951	16	373.63
17	8	-0.687158	0.030019	2.440512	17	419.49
18	1	-1.398472	0.641087	2.193686	18	433.49
19	7	2.298859	-1.393288	-0.355137	19	449.11
20	8	3.469148	-1.647830	-0.591285	20	518.47
21	8	1.431335	-2.246114	-0.247942	21	549.94
22	6	-2.302204	0.014219	-0.062019	22	576.11
23	8	-2.438794	1.073280	0.530825	23	603.93
24	8	-3.033266	-0.321676	-1.118442	24	621.34
25	6	-4.048168	0.617047	-1.508848	25	669.46
26	1	-4.533873	0.174182	-2.374970	26	705.86
27	1	-4.760951	0.756209	-0.694584	27	720.30
28	1	-3.590892	1.572931	-1.768597	28	730.71
					29	764.00
					30	815.31
					31	837.03
					32	852.21
					33	883.12
					34	888.18
					35	907.34
					36	966.41
					37	997.04

38	1029.38
39	1037.12
40	1046.89
41	1087.58
42	1094.97
43	1126.57
44	1180.55
45	1184.41
46	1185.48
47	1202.45
48	1216.71
49	1236.57
50	1287.71
51	1293.69
52	1333.91
53	1365.06
54	1393.08
55	1435.09
56	1456.42
57	1469.40
58	1482.19
59	1490.41
60	1492.21
61	1495.19
62	1529.71
63	1647.43
64	1666.51
65	1700.35
66	1724.22
67	1780.47
68	3093.66
69	3173.60
70	3183.30
71	3195.44
72	3217.85
73	3218.80
74	3234.20
75	3251.68
76	3254.18
77	3293.60
78	3779.40

-- ------------------

```
Sum of Electronic and Nuclear Energy:            -856.254845 (Hartree)
Sum of Electronic and Thermal Free Energies:     -856.083463
Standard Free Energy (1,0 M):                    -856.080444
===================================================================
===================================================================
```

Structure: 2a (Charge = -1)

Center Number	Atomic Symbol	X	Y	Z	Normal Mode	Frequencies (cm**-1)
1	6	1.783809	-0.059954	-0.205615	1	28.07
2	6	2.536781	0.778068	-1.052937	2	39.66
3	6	2.405074	2.157783	-0.988548	3	71.65
4	6	1.512576	2.732703	-0.082796	4	75.07
5	6	0.732793	1.901369	0.722293	5	113.64
6	6	0.826799	0.509878	0.678275	6	129.82
7	1	3.237046	0.312788	-1.734926	7	159.25
8	1	3.010539	2.783734	-1.637403	8	175.39
9	1	1.408257	3.810591	-0.013028	9	189.85
10	1	0.015721	2.351588	1.400307	10	225.50
11	6	-0.183361	-0.332954	1.460422	11	242.66
12	6	-1.189148	-0.936093	0.484589	12	283.01
13	1	0.335765	-1.155105	1.945900	13	295.62
14	6	-1.197345	-2.229563	0.151369	14	351.17
15	1	-1.916719	-2.621661	-0.559593	15	363.91
16	1	-0.459828	-2.901573	0.578259	16	371.51
17	8	-0.833864	0.396525	2.489429	17	377.75
18	1	-1.458251	1.005840	2.066424	18	426.25
19	7	2.037120	-1.440053	-0.274389	19	449.45
20	8	2.656077	-1.911464	-1.305143	20	503.38
21	8	1.801652	-2.194516	0.747512	21	538.89
22	6	-2.184959	0.008250	-0.101700	22	563.46
23	8	-2.366901	1.140441	0.319576	23	566.21
24	8	-2.875508	-0.492854	-1.124248	24	600.83
25	6	-3.888582	0.358174	-1.678484	25	614.17
26	1	-4.351137	-0.220537	-2.474642	26	653.45
27	1	-4.622065	0.615181	-0.912306	27	678.33
28	1	-3.437440	1.268495	-2.076492	28	711.77
					29	746.93
					30	766.60
					31	803.01
					32	837.51

33	851.09
34	877.89
35	885.99
36	962.59
37	974.98
38	1009.02
39	1030.59
40	1049.51
41	1074.95
42	1082.85
43	1107.81
44	1152.00
45	1172.37
46	1181.01
47	1198.32
48	1201.73
49	1233.67
50	1279.62
51	1292.35
52	1302.24
53	1346.84
54	1364.68
55	1383.43
56	1414.12
57	1418.24
58	1455.09
59	1489.02
60	1489.54
61	1494.96
62	1495.06
63	1524.46
64	1629.89
65	1663.17
66	1723.76
67	1775.63
68	3093.12
69	3181.08
70	3196.72
71	3204.86
72	3213.24
73	3214.24
74	3227.45

					75	3237.62
					76	3257.33
					77	3297.17
					78	3791.04

Sum of Electronic and Nuclear Energy: -856.380620 (Hartree)
Sum of Electronic and Thermal Free Energies: -856.212176
Standard Free Energy (1,0 M): -856.209157

Structure: 2b (Charge = 0)

Center Number	Atomic Symbol	Coordinates (Angstroms) X	Y	Z	Normal Mode	Frequencies (cm**-1)
1	6	-2.422141	0.083445	-0.207233	1	12.47
2	6	-2.475962	0.866516	-1.353095	2	36.05
3	6	-1.386747	1.689408	-1.625179	3	48.79
4	6	-0.287297	1.705689	-0.769421	4	66.51
5	6	-0.252769	0.903570	0.377158	5	102.03
6	6	-1.339752	0.084344	0.665842	6	143.18
7	6	0.957335	0.952416	1.308332	7	152.10
8	6	2.249531	0.770393	0.533481	8	170.13
9	6	3.165218	1.731069	0.399081	9	180.58
10	6	2.436986	-0.583093	-0.067180	10	182.21
11	8	0.852285	0.033534	2.376687	11	210.58
12	7	-3.564445	-0.789029	0.099675	12	280.70
13	8	-3.521793	-1.456430	1.120647	13	303.81
14	8	-4.501491	-0.804745	-0.682487	14	340.56
15	8	3.467901	-0.672756	-0.898952	15	369.20
16	8	1.704208	-1.522813	0.195605	16	408.18
17	6	3.708996	-1.968429	-1.470100	17	414.13
18	1	-3.341597	0.832112	-2.002386	18	431.90
19	1	-1.395729	2.317819	-2.508700	19	471.96
20	1	0.557669	2.349589	-0.998517	20	501.54
21	1	-1.352341	-0.537981	1.551704	21	536.41
22	1	0.987132	1.944599	1.770940	22	554.56
23	1	4.074552	1.566972	-0.168972	23	596.58
24	1	3.020249	2.704702	0.858176	24	607.60
25	1	0.941102	-0.856688	2.000260	25	663.83
26	1	4.587031	-1.848674	-2.100181	26	682.80
27	1	2.847661	-2.279343	-2.062993	27	703.48

28	1	3.896121	-2.696196	-0.679166	28	745.23
--					29	768.43
					30	826.32
					31	842.84
					32	853.68
					33	874.81
					34	928.17
					35	957.58
					36	970.53
					37	990.78
					38	1020.31
					39	1030.28
					40	1030.94
					41	1045.71
					42	1106.81
					43	1136.14
					44	1141.94
					45	1184.88
					46	1199.69
					47	1205.60
					48	1229.61
					49	1242.07
					50	1284.31
					51	1301.54
					52	1324.29
					53	1375.91
					54	1394.64
					55	1421.47
					56	1452.75
					57	1467.81
					58	1486.24
					59	1490.56
					60	1492.75
					61	1498.18
					62	1538.12
					63	1652.79
					64	1673.51
					65	1714.90
					66	1734.01
					67	1787.36
					68	3100.95
					69	3114.86

70	3187.59
71	3192.07
72	3213.88
73	3217.10
74	3249.52
75	3270.86
76	3279.66
77	3283.74
78	3792.78

-- ------------------

Sum of Electronic and Nuclear Energy: -856.262691 (Hartree)

Sum of Electronic and Thermal Free Energies: -856.092763

Standard Free Energy (1,0 M): -856.089744

===

===

Structure: 2b (Charge = -1)

Center Number	Atomic Symbol	Coordinates (Angstroms) X	Y	Z	Normal Mode	Frequencies (cm**-1)
1	6	-2.498800	-0.207862	0.101595	1	14.45
2	6	-2.442172	-1.588099	0.376084	2	34.13
3	6	-1.298444	-2.298799	0.035358	3	61.41
4	6	-0.207208	-1.667410	-0.567771	4	86.47
5	6	-0.264720	-0.292534	-0.839265	5	111.98
6	6	-1.402962	0.433916	-0.510772	6	149.44
7	6	0.909363	0.393895	-1.536575	7	153.24
8	6	2.240512	-0.064948	-0.971864	8	165.62
9	6	3.134216	-0.761060	-1.676714	9	182.68
10	6	2.500872	0.336742	0.441893	10	191.42
11	8	0.806847	1.806990	-1.520120	11	210.65
12	7	-3.636193	0.525371	0.433687	12	282.63
13	8	-3.672559	1.789713	0.183865	13	299.09
14	8	-4.635307	-0.072133	0.988163	14	344.71
15	8	3.556128	-0.259311	0.991138	15	368.94
16	8	1.810440	1.147314	1.036522	16	372.86
17	6	3.868805	0.128201	2.337381	17	408.23
18	1	-3.289833	-2.068543	0.847596	18	415.84
19	1	-1.252591	-3.363510	0.245822	19	454.34
20	1	0.679269	-2.239461	-0.824737	20	491.19
21	1	-1.468089	1.492986	-0.721988	21	520.96
22	1	0.891567	0.113683	-2.595522	22	534.33

23	1	4.075802	-1.080208	-1.242887
24	1	2.934386	-1.026880	-2.710864
25	1	0.859184	2.081942	-0.591396
26	1	4.751150	-0.446841	2.607734
27	1	3.034315	-0.112112	2.997935
28	1	4.077019	1.198505	2.379847

23	552.03
24	604.20
25	619.16
26	659.87
27	676.92
28	706.60
29	725.28
30	780.04
31	814.05
32	844.82
33	859.98
34	903.96
35	904.95
36	932.76
37	993.01
38	1001.74
39	1004.08
40	1022.28
41	1047.14
42	1089.05
43	1108.43
44	1123.12
45	1178.09
46	1184.15
47	1209.71
48	1214.86
49	1238.22
50	1275.19
51	1299.79
52	1310.08
53	1351.12
54	1370.97
55	1391.89
56	1418.83
57	1428.45
58	1456.31
59	1490.99
60	1492.91
61	1497.23
62	1503.14
63	1532.18
64	1640.58

65	1661.03
66	1729.33
67	1783.91
68	3097.53
69	3106.18
70	3182.98
71	3183.92
72	3200.96
73	3227.12
74	3227.82
75	3258.66
76	3259.12
77	3281.94
78	3793.79

Sum of Electronic and Nuclear Energy: -856.387994 (Hartree)

Sum of Electronic and Thermal Free Energies: -856.220978

Standard Free Energy (1,0 M): -856.217959

Structure: 2c (Charge = 0)

Center Number	Atomic Symbol	Coordinates (Angstroms) X	Y	Z	Normal Mode	Frequencies (cm**-1)
1	6	-2.216187	-0.787286	-0.929821	1	20.37
2	6	-2.640379	0.078608	0.070553	2	30.04
3	6	-1.837151	0.418584	1.154279	3	54.61
4	6	-0.562371	-0.128312	1.225182	4	57.84
5	6	-0.100982	-1.000241	0.231529	5	84.67
6	6	-0.937572	-1.327147	-0.838600	6	112.60
7	6	1.290406	-1.619188	0.346774	7	134.25
8	6	2.330013	-0.584838	0.735297	8	149.49
9	6	2.983159	-0.601473	1.898328	9	178.54
10	6	2.591474	0.457529	-0.300351	10	193.50
11	8	1.664954	-2.321157	-0.821713	11	261.98
12	7	-3.985613	0.654986	-0.016693	12	284.21
13	8	-4.674937	0.363744	-0.981500	13	294.75
14	8	-4.352286	1.398408	0.879950	14	330.78
15	8	3.373298	1.449574	0.108487	15	351.27
16	8	2.127375	0.389643	-1.426747	16	378.65
17	6	3.686078	2.451963	-0.871415	17	418.43

18	1	-2.872321	-1.031095	-1.756296
19	1	-2.204715	1.095276	1.915803
20	1	0.078505	0.129735	2.062828
21	1	-0.588536	-2.011484	-1.603117
22	1	1.255298	-2.369034	1.144732
23	1	3.719187	0.157261	2.141557
24	1	2.792217	-1.379181	2.632116
25	1	1.788363	-1.664051	-1.525378
26	1	2.771081	2.941233	-1.208681
27	1	4.196833	1.995649	-1.720663
28	1	4.337219	3.159883	-0.364399

18	457.03
19	497.37
20	508.96
21	530.62
22	548.20
23	594.10
24	619.06
25	638.83
26	680.58
27	715.56
28	738.66
29	765.85
30	829.33
31	847.27
32	868.82
33	876.65
34	888.92
35	911.53
36	965.73
37	1009.71
38	1023.84
39	1028.36
40	1033.03
41	1044.72
42	1113.19
43	1136.15
44	1147.41
45	1182.27
46	1203.30
47	1204.47
48	1222.36
49	1238.21
50	1280.43
51	1302.64
52	1321.63
53	1374.11
54	1393.05
55	1416.92
56	1445.80
57	1460.76
58	1464.08
59	1485.11

					60	1487.22
					61	1497.20
					62	1541.72
					63	1644.11
					64	1683.64
					65	1702.34
					66	1728.67
					67	1786.20
					68	3098.35
					69	3113.39
					70	3182.78
					71	3189.19
					72	3217.86
					73	3222.98
					74	3242.47
					75	3256.03
					76	3260.28
					77	3279.23
					78	3799.67

Sum of Electronic and Nuclear Energy: -856.262818 (Hartree)

Sum of Electronic and Thermal Free Energies: -856.093543

Standard Free Energy (1,0 M): -856.090524

Structure: 2c (Charge = -1)

Center Number	Atomic Symbol	Coordinates (Angstroms) X	Y	Z	Normal Mode	Frequencies (cm**-1)
1	6	-2.206495	-0.785247	-0.930355	1	25.38
2	6	-2.680256	0.087965	0.067030	2	29.78
3	6	-1.846989	0.404655	1.158683	3	60.12
4	6	-0.572894	-0.140939	1.232878	4	82.05
5	6	-0.088587	-1.009156	0.243868	5	109.24
6	6	-0.928108	-1.321833	-0.830446	6	113.12
7	6	1.302526	-1.622136	0.368226	7	150.44
8	6	2.338239	-0.576226	0.737726	8	154.66
9	6	2.985126	-0.564993	1.904789	9	176.78
10	6	2.605124	0.451383	-0.311741	10	190.44
11	8	1.689466	-2.335344	-0.794746	11	256.19
12	7	-3.958800	0.633322	-0.022783	12	281.23

13	8	-4.702140	0.342111	-1.035827
14	8	-4.380205	1.421640	0.907484
15	8	3.334188	1.481708	0.109047
16	8	2.198407	0.348742	-1.457064
17	6	3.656459	2.470619	-0.879847
18	1	-2.850529	-1.027971	-1.766089
19	1	-2.214957	1.076495	1.923845
20	1	0.058956	0.116582	2.079777
21	1	-0.577886	-2.001136	-1.601249
22	1	1.291709	-2.366152	1.173043
23	1	3.712320	0.204013	2.142139
24	1	2.795557	-1.332871	2.649451
25	1	1.772674	-1.684558	-1.509613
26	1	2.741511	2.913045	-1.276799
27	1	4.228082	2.015781	-1.690454
28	1	4.251553	3.218095	-0.360599

13	286.36
14	332.85
15	351.15
16	375.12
17	388.43
18	430.33
19	489.15
20	497.30
21	521.10
22	532.95
23	547.65
24	592.80
25	619.51
26	641.98
27	674.08
28	712.68
29	716.82
30	794.65
31	827.86
32	844.06
33	847.98
34	865.20
35	889.24
36	964.38
37	984.15
38	1002.67
39	1018.51
40	1019.43
41	1046.68
42	1097.22
43	1104.15
44	1125.39
45	1181.85
46	1188.68
47	1204.43
48	1222.29
49	1235.01
50	1277.41
51	1300.12
52	1305.15
53	1334.11
54	1376.99

					55	1389.84
					56	1417.22
					57	1440.46
					58	1447.09
					59	1464.46
					60	1483.03
					61	1487.55
					62	1499.06
					63	1550.63
					64	1632.62
					65	1669.25
					66	1726.26
					67	1786.68
					68	3098.04
					69	3098.40
					70	3181.44
					71	3188.73
					72	3202.14
					73	3216.45
					74	3217.83
					75	3253.76
					76	3255.57
					77	3277.22
					78	3795.41

Sum of Electronic and Nuclear Energy: -856.387543 (Hartree)
Sum of Electronic and Thermal Free Energies: -856.220791
Standard Free Energy (1,0 M): -856.217772

Group 3

Structure: 3a (Charge = 0)

Center Number	Atomic Symbol	Coordinates (Angstroms) X	Y	Z	Normal Mode	Frequencies (cm**-1)
1	6	2.457955	0.191777	-0.237419	1	15.50
2	6	3.161824	1.196748	-0.901414	2	38.17
3	6	2.742872	2.513778	-0.790771	3	42.28
4	6	1.639081	2.808196	0.006446	4	53.55

5	6	0.958561	1.792087	0.670335
6	6	1.328269	0.446567	0.561197
7	1	3.277529	3.298977	-1.313090
8	1	1.303369	3.833980	0.117690
9	1	0.115643	2.054746	1.295991
10	6	0.516731	-0.603471	1.338949
11	6	-0.539518	-1.276247	0.468906
12	1	1.191619	-1.379554	1.697655
13	6	-0.563156	-2.587979	0.223676
14	1	-1.358568	-3.033835	-0.363809
15	1	0.212614	-3.239348	0.610904
16	8	-0.070043	-0.042665	2.499182
17	1	-0.855598	0.458259	2.232067
18	6	-1.651583	-0.407123	-0.007395
19	8	-1.894520	0.682923	0.498534
20	8	-2.358763	-0.921029	-1.000226
21	6	-3.458039	-0.145892	-1.535501
22	1	-3.078173	0.824158	-1.869259
23	1	-3.770709	-0.732316	-2.398591
24	6	-4.616966	0.038330	-0.559507
25	1	-4.637480	-0.813411	0.134890
26	1	-5.548877	0.024964	-1.129831
27	8	-4.599571	1.272956	0.131056
28	1	-3.722214	1.366019	0.530747
29	1	4.025979	0.930633	-1.496502
30	7	2.998132	-1.164449	-0.408178
31	8	2.286133	-2.114970	-0.123933
32	8	4.136983	-1.282560	-0.831693

5	82.58
6	88.37
7	120.34
8	142.28
9	162.25
10	202.66
11	216.19
12	235.70
13	273.70
14	290.03
15	315.48
16	353.95
17	371.75
18	381.54
19	424.85
20	429.75
21	449.45
22	475.37
23	501.42
24	518.04
25	561.44
26	571.98
27	609.87
28	624.06
29	668.11
30	704.03
31	715.27
32	728.90
33	767.80
34	817.36
35	820.98
36	841.18
37	850.37
38	887.03
39	890.20
40	922.23
41	966.26
42	977.94
43	1011.14
44	1028.93
45	1039.66
46	1056.79

47	1087.53
48	1092.79
49	1099.26
50	1124.25
51	1140.65
52	1185.42
53	1189.92
54	1213.43
55	1217.97
56	1238.69
57	1274.49
58	1298.03
59	1308.88
60	1334.50
61	1363.52
62	1373.08
63	1391.32
64	1423.90
65	1430.90
66	1451.08
67	1458.61
68	1465.33
69	1489.29
70	1494.11
71	1499.65
72	1535.67
73	1647.27
74	1667.80
75	1702.45
76	1718.20
77	1764.43
78	3049.57
79	3117.76
80	3136.39
81	3164.32
82	3190.04
83	3197.20
84	3219.89
85	3252.19
86	3272.55
87	3281.78
88	3287.17

					89	3804.26
					90	3826.33

Sum of Electronic and Nuclear Energy: -970.750074 (Hartree)
Sum of Electronic and Thermal Free Energies: -970.547524
Standard Free Energy (1,0 M): -970.544505

Structure: 3a (Charge = -1)

Center Number	Atomic Symbol	Coordinates (Angstroms) X	Y	Z	Normal Mode	Frequencies (cm**-1)
1	6	2.312033	0.135363	-0.291473	1	22.65
2	6	2.875700	1.067914	-1.184847	2	41.09
3	6	2.578655	2.419416	-1.080936	3	47.71
4	6	1.705361	2.868059	-0.089287	4	58.94
5	6	1.108290	1.937950	0.762400	5	77.28
6	6	1.371204	0.570411	0.680282	6	84.56
7	1	3.039829	3.123497	-1.767228	7	118.28
8	1	1.474642	3.923546	0.011717	8	155.08
9	1	0.402979	2.288318	1.508310	9	156.58
10	6	0.552334	-0.406320	1.526471	10	206.81
11	6	-0.406991	-1.175389	0.623642	11	222.36
12	1	1.220159	-1.132915	1.983142	12	232.79
13	6	-0.259318	-2.471251	0.334763	13	280.64
14	1	-0.954028	-2.983985	-0.321992	14	287.75
15	1	0.582430	-3.020905	0.743262	15	317.31
16	8	-0.137795	0.227327	2.591998	16	354.35
17	1	-0.861666	0.745625	2.209809	17	361.89
18	6	-1.547950	-0.398881	0.062855	18	371.32
19	8	-1.845871	0.722974	0.458346	19	383.20
20	8	-2.222476	-1.025293	-0.892922	20	429.08
21	6	-3.315931	-0.327794	-1.532241	21	442.62
22	1	-2.949281	0.625331	-1.924809	22	476.77
23	1	-3.576963	-0.986905	-2.359693	23	497.76
24	6	-4.522536	-0.105191	-0.625720	24	507.14
25	1	-4.566226	-0.916869	0.114213	25	536.60
26	1	-5.425403	-0.164049	-1.238804	26	560.21
27	8	-4.553700	1.165895	-0.004252	27	568.05
28	1	-3.683860	1.308737	0.398576	28	607.48
29	1	3.566790	0.699686	-1.932558	29	613.48

30	7	2.729884	-1.201399	-0.403172
31	8	2.719284	-1.969503	0.635805
32	8	3.280096	-1.594529	-1.503624

30	652.94
31	678.71
32	703.65
33	749.55
34	763.97
35	801.43
36	821.50
37	841.59
38	851.36
39	887.83
40	889.07
41	957.52
42	973.70
43	977.75
44	1006.58
45	1017.55
46	1059.25
47	1077.31
48	1086.57
49	1094.30
50	1108.38
51	1141.39
52	1156.04
53	1173.17
54	1202.35
55	1219.42
56	1243.47
57	1279.84
58	1297.41
59	1307.15
60	1308.97
61	1349.36
62	1367.79
63	1369.90
64	1391.67
65	1416.64
66	1418.57
67	1430.25
68	1451.31
69	1457.37
70	1490.29
71	1496.76

					72	1497.24
					73	1528.89
					74	1635.08
					75	1667.33
					76	1711.68
					77	1758.22
					78	3058.09
					79	3116.81
					80	3143.18
					81	3181.26
					82	3185.14
					83	3199.76
					84	3204.82
					85	3219.57
					86	3235.22
					87	3260.67
					88	3283.28
					89	3807.48
					90	3812.17

Sum of Electronic and Nuclear Energy: -970.876666 (Hartree)

Sum of Electronic and Thermal Free Energies: -970.677142

Standard Free Energy (1,0 M): -970.674123

Structure: 3b (Charge = 0)

Center Number	Atomic Symbol	Coordinates (Angstroms) X	Y	Z	Normal Mode	Frequencies (cm**-1)
1	6	1.693225	0.046696	0.547710	1	16.13
2	6	2.775645	-0.260197	-0.266764	2	27.87
3	6	3.206775	0.565007	-1.298680	3	42.27
4	6	2.511725	1.750916	-1.508628	4	45.10
5	6	1.421591	2.084879	-0.703884	5	75.70
6	6	1.001750	1.237076	0.324442	6	89.58
7	1	4.054953	0.283341	-1.909794	7	94.29
8	1	2.821700	2.419921	-2.303853	8	143.35
9	1	0.891941	3.016198	-0.873173	9	162.61
10	6	-0.166562	1.620489	1.232018	10	182.77
11	6	-1.051858	0.427102	1.539724	11	204.96
12	1	0.247954	1.974039	2.181975	12	213.38

13	6	-1.155102	-0.113695	2.755237	13	269.76
14	1	-1.790547	-0.972919	2.940655	14	297.49
15	1	-0.598897	0.297719	3.592656	15	311.12
16	8	-0.908722	2.706999	0.715407	16	356.79
17	1	-1.344581	2.400797	-0.095408	17	374.88
18	6	-1.822126	-0.104182	0.379686	18	381.40
19	8	-1.828486	0.462146	-0.706428	19	426.95
20	8	-2.494444	-1.216048	0.628244	20	428.72
21	6	-3.236590	-1.827557	-0.454368	21	468.31
22	1	-2.556310	-2.013333	-1.290731	22	492.82
23	1	-3.556870	-2.776289	-0.025105	23	499.41
24	6	-4.442628	-1.011300	-0.908978	24	516.67
25	1	-4.820654	-0.431593	-0.055062	25	552.25
26	1	-5.228630	-1.706857	-1.213517	26	560.27
27	8	-4.196259	-0.184704	-2.030488	27	600.53
28	1	-3.394428	0.326432	-1.843879	28	616.38
29	1	1.402391	-0.637270	1.337332	29	664.03
30	7	3.494746	-1.519044	-0.026562	30	684.79
31	8	4.447204	-1.781351	-0.743239	31	706.83
32	8	3.104917	-2.241827	0.876348	32	741.83
---					33	766.42
					34	818.87
					35	826.89
					36	843.35
					37	853.28
					38	885.07
					39	925.04
					40	945.55
					41	971.55
					42	975.58
					43	981.82
					44	1020.30
					45	1027.87
					46	1029.36
					47	1056.84
					48	1093.45
					49	1124.38
					50	1132.09
					51	1142.22
					52	1144.45
					53	1194.91
					54	1229.53

55	1237.46
56	1243.22
57	1281.32
58	1304.46
59	1311.59
60	1326.91
61	1372.38
62	1376.56
63	1393.29
64	1421.39
65	1433.03
66	1451.62
67	1456.42
68	1468.38
69	1489.94
70	1490.55
71	1497.37
72	1539.47
73	1654.79
74	1673.06
75	1715.30
76	1724.65
77	1768.22
78	3061.39
79	3113.77
80	3115.11
81	3140.34
82	3179.06
83	3193.04
84	3222.16
85	3236.11
86	3236.92
87	3276.19
88	3278.58
89	3796.43
90	3821.27

```
------------------------------------------------ -----------------
Sum of Electronic and Nuclear Energy:            -970.758098 (Hartree)
Sum of Electronic and Thermal Free Energies:     -970.556852
Standard Free Energy (1,0 M):                    -970.553833
==================================================================
==================================================================
```

Structure: 3b (Charge = -1)

Center Number	Atomic Symbol	Coordinates (Angstroms) X	Y	Z	Normal Mode	Frequencies (cm**-1)
1	6	1.677469	0.064679	0.542275	1	9.11
2	6	2.781078	-0.304688	-0.249798	2	28.86
3	6	3.202207	0.533947	-1.300847	3	40.65
4	6	2.520024	1.719802	-1.533296	4	76.22
5	6	1.425062	2.095266	-0.746399	5	86.90
6	6	1.004032	1.258531	0.292631	6	92.61
7	1	4.049680	0.237283	-1.905436	7	101.48
8	1	2.844448	2.368475	-2.342014	8	142.77
9	1	0.908011	3.029257	-0.935581	9	160.69
10	6	-0.162072	1.665057	1.192586	10	176.77
11	6	-1.054912	0.484569	1.524835	11	198.61
12	1	0.246160	2.039134	2.137653	12	213.24
13	6	-1.181270	-0.016775	2.754982	13	266.92
14	1	-1.821645	-0.867939	2.959518	14	295.77
15	1	-0.636850	0.421362	3.586572	15	311.14
16	8	-0.913092	2.742039	0.658551	16	352.57
17	1	-1.310853	2.428797	-0.168522	17	368.84
18	6	-1.813610	-0.081147	0.373639	18	376.17
19	8	-1.845663	0.468337	-0.720437	19	381.44
20	8	-2.454961	-1.209557	0.640083	20	426.04
21	6	-3.189251	-1.851959	-0.428517	21	451.67
22	1	-2.510759	-2.033818	-1.267359	22	476.01
23	1	-3.484953	-2.802294	0.015017	23	504.25
24	6	-4.417434	-1.071396	-0.887221	24	517.19
25	1	-4.805848	-0.490802	-0.038434	25	522.34
26	1	-5.187678	-1.789483	-1.179967	26	540.77
27	8	-4.195515	-0.251816	-2.019064	27	559.19
28	1	-3.399440	0.272411	-1.842293	28	604.17
29	1	1.372063	-0.597929	1.343487	29	624.97
30	7	3.451224	-1.499548	0.004624	30	661.11
31	8	4.460199	-1.827547	-0.728098	31	680.48
32	8	3.052928	-2.258072	0.968437	32	707.03
					33	719.53
					34	777.17
					35	811.52
					36	824.45
					37	844.72

38	869.12
39	888.71
40	902.36
41	928.81
42	971.02
43	982.60
44	998.90
45	1003.68
46	1023.08
47	1058.44
48	1092.93
49	1100.99
50	1116.08
51	1121.30
52	1142.35
53	1181.43
54	1218.33
55	1227.24
56	1242.47
57	1277.03
58	1299.32
59	1304.19
60	1318.93
61	1351.20
62	1370.54
63	1375.70
64	1392.38
65	1421.71
66	1427.05
67	1433.08
68	1451.73
69	1457.07
70	1487.71
71	1496.58
72	1500.85
73	1540.64
74	1648.14
75	1664.69
76	1724.79
77	1769.11
78	3059.84
79	3107.98

80	3111.74
81	3140.35
82	3181.12
83	3189.20
84	3199.42
85	3229.54
86	3238.57
87	3268.19
88	3279.64
89	3795.38
90	3811.32

Sum of Electronic and Nuclear Energy: -970.884067 (Hartree)
Sum of Electronic and Thermal Free Energies: -970.686352
Standard Free Energy (1,0 M): -970.683333

Structure: 3c (Charge = 0)

Center Number	Atomic Symbol	X	Y	Z	Normal Mode	Frequencies (cm**-1)
1	6	-2.474255	0.282926	-1.164698	1	23.89
2	6	-3.125742	-0.255826	-0.060079	2	31.71
3	6	-2.651526	-0.098440	1.236309	3	38.83
4	6	-1.477481	0.622354	1.428751	4	58.48
5	6	-0.793259	1.169814	0.341024	5	73.90
6	6	-1.302610	0.997288	-0.951850	6	85.07
7	6	0.479424	1.987326	0.549651	7	102.76
8	6	1.563545	1.586835	-0.433902	8	110.55
9	6	2.013753	2.392353	-1.397685	9	154.73
10	6	2.113170	0.214706	-0.243114	10	185.31
11	8	0.938987	1.942103	1.884899	11	201.84
12	8	2.944163	-0.180442	-1.193163	12	261.34
13	8	1.813619	-0.473279	0.725102	13	276.57
14	6	3.480521	-1.523427	-1.124462	14	290.04
15	6	4.457156	-1.738715	0.027622	15	304.80
16	8	3.873855	-2.310904	1.182580	16	340.27
17	1	-3.190028	-0.531217	2.070539	17	374.67
18	1	-1.093853	0.763115	2.432612	18	381.47
19	1	0.234398	3.037327	0.356595	19	416.82
20	1	2.785120	2.068890	-2.088192	20	451.36

21	1	1.613418	3.395183	-1.513509
22	1	1.243272	1.037323	2.057554
23	1	3.987671	-1.632613	-2.082476
24	1	2.650605	-2.233732	-1.064995
25	1	4.954839	-0.785604	0.255538
26	1	5.223501	-2.443529	-0.304177
27	1	3.087431	-1.788690	1.399422
28	1	-0.779920	1.423830	-1.802717
29	1	-2.878000	0.143270	-2.159839
30	8	-4.932285	-1.471878	0.705889
31	8	-4.763169	-1.152397	-1.417319
32	7	-4.362283	-1.014622	-0.272238

21	469.00
22	484.83
23	507.36
24	514.06
25	543.59
26	565.63
27	607.12
28	620.68
29	638.88
30	684.75
31	712.44
32	737.62
33	765.92
34	818.17
35	836.94
36	848.31
37	862.15
38	883.48
39	892.79
40	910.45
41	969.49
42	982.05
43	1005.06
44	1019.11
45	1028.85
46	1034.49
47	1059.52
48	1093.27
49	1116.76
50	1139.44
51	1142.11
52	1148.76
53	1205.48
54	1224.12
55	1231.73
56	1240.16
57	1283.05
58	1303.62
59	1312.05
60	1324.18
61	1374.27
62	1377.98

					63	1390.27
					64	1424.66
					65	1432.39
					66	1451.22
					67	1458.18
					68	1466.03
					69	1469.46
					70	1488.90
					71	1500.36
					72	1543.61
					73	1649.15
					74	1686.05
					75	1706.76
					76	1727.38
					77	1775.60
					78	3054.81
					79	3109.72
					80	3113.83
					81	3139.51
					82	3185.51
					83	3189.99
					84	3215.17
					85	3243.22
					86	3255.44
					87	3268.03
					88	3283.37
					89	3791.51
					90	3835.16

Sum of Electronic and Nuclear Energy: -970.758467 (Hartree)

Sum of Electronic and Thermal Free Energies: -970.556821

Standard Free Energy (1,0 M): -970.553802

Structure: 3c (Charge = -1)

Center Number	Atomic Symbol	Coordinates (Angstroms) X	Y	Z	Normal Mode	Frequencies (cm**-1)
1	6	-2.497963	0.300574	-1.153681	1	22.37
2	6	-3.172924	-0.275321	-0.058566	2	33.57
3	6	-2.648152	-0.112679	1.237571	3	43.09

4	6	-1.473700	0.607282	1.423977	4	71.04
5	6	-0.790664	1.176710	0.344497	5	85.08
6	6	-1.325468	1.011501	-0.941458	6	103.55
7	6	0.481566	1.992859	0.545972	7	109.29
8	6	1.564016	1.579099	-0.434633	8	113.14
9	6	2.005892	2.369229	-1.415436	9	155.72
10	6	2.120963	0.211064	-0.233126	10	178.72
11	8	0.953334	1.952015	1.881796	11	206.36
12	8	2.913386	-0.206710	-1.209517	12	256.83
13	8	1.869578	-0.458512	0.761026	13	273.66
14	6	3.458803	-1.544010	-1.127248	14	281.54
15	6	4.484812	-1.725090	-0.012340	15	307.67
16	8	3.952863	-2.266095	1.181848	16	340.52
17	1	-3.172373	-0.553924	2.075872	17	372.23
18	1	-1.084620	0.733029	2.429555	18	375.88
19	1	0.258676	3.047936	0.350960	19	395.87
20	1	2.768112	2.037017	-2.111897	20	429.83
21	1	1.604468	3.370892	-1.539108	21	472.11
22	1	1.221084	1.037361	2.061736	22	475.89
23	1	3.925754	-1.678334	-2.102351	23	500.14
24	1	2.636842	-2.256953	-1.012367	24	511.10
25	1	4.987487	-0.764397	0.168056	25	521.42
26	1	5.239433	-2.436552	-0.356792	26	539.05
27	1	3.169626	-1.743537	1.410645	27	566.05
28	1	-0.814421	1.448243	-1.796471	28	607.07
29	1	-2.907004	0.176062	-2.148377	29	620.89
30	8	-4.951479	-1.520230	0.753664	30	641.87
31	8	-4.817259	-1.125329	-1.453024	31	675.33
32	7	-4.348506	-0.995037	-0.258425	32	711.71
					33	716.10
					34	793.27
					35	821.76
					36	829.60
					37	844.55
					38	845.43
					39	872.01
					40	895.53
					41	970.07
					42	978.65
					43	984.66
					44	1000.37
					45	1020.23

46	1021.38
47	1061.50
48	1094.88
49	1096.68
50	1108.47
51	1128.19
52	1141.05
53	1191.46
54	1223.20
55	1228.64
56	1241.23
57	1280.68
58	1304.03
59	1307.54
60	1311.63
61	1336.10
62	1375.13
63	1378.22
64	1391.40
65	1425.37
66	1432.01
67	1441.25
68	1452.32
69	1460.90
70	1469.17
71	1491.60
72	1501.93
73	1549.79
74	1633.62
75	1670.52
76	1723.87
77	1773.00
78	3054.26
79	3094.34
80	3109.91
81	3138.94
82	3184.67
83	3192.36
84	3194.29
85	3222.45
86	3250.03
87	3266.24

					88	3282.20
					89	3798.79
					90	3832.89

Sum of Electronic and Nuclear Energy: -970.883413 (Hartree)
Sum of Electronic and Thermal Free Energies: -970.684533
Standard Free Energy (1,0 M): -970.681514

Group 4

Structure: 4a (Charge = 0)

Center Number	Atomic Symbol	Coordinates (Angstroms) X	Y	Z	Normal Mode	Frequencies (cm**-1)
1	6	2.596911	0.157148	-0.290346	1	18.37
2	6	3.325010	1.164746	-0.922463	2	34.87
3	6	2.942215	2.487517	-0.759388	3	38.55
4	6	1.854383	2.782119	0.060107	4	54.52
5	6	1.154092	1.761787	0.696517	5	71.64
6	6	1.486571	0.411920	0.532347	6	82.05
7	1	1.548409	3.812150	0.211251	7	104.71
8	1	0.325274	2.021849	1.342714	8	131.53
9	6	0.666081	-0.646577	1.286713	9	157.22
10	6	-0.485578	-1.180884	0.443118	10	188.89
11	1	1.310828	-1.488834	1.534993	11	221.41
12	6	-0.585010	-2.455916	0.061873	12	229.94
13	1	-1.440573	-2.802230	-0.508035	13	238.63
14	1	0.187014	-3.174625	0.314655	14	264.73
15	8	0.201138	-0.141946	2.525394	15	284.71
16	1	-0.580607	0.406175	2.353190	16	297.95
17	6	-1.593649	-0.223098	0.152879	17	341.21
18	8	-1.780626	0.789044	0.811547	18	355.26
19	8	-2.360476	-0.582430	-0.869940	19	374.21
20	6	-3.526131	0.227979	-1.138638	20	415.38
21	6	-4.675125	-0.139779	-0.215704	21	421.62
22	1	-3.769915	0.011426	-2.180138	22	441.86
23	1	-3.253960	1.282113	-1.040742	23	494.50
24	6	-5.923318	0.672365	-0.558045	24	522.29
25	1	-4.878816	-1.211194	-0.316861	25	571.67

26	1	-4.377078	0.044463	0.821644	26	576.26
27	1	-6.754589	0.400565	0.096518	27	618.12
28	1	-5.738450	1.744646	-0.440278	28	624.76
29	1	-6.236557	0.494535	-1.591713	29	670.19
30	1	4.175779	0.896306	-1.536163	30	705.32
31	1	3.491689	3.276918	-1.259579	31	719.87
32	7	3.087119	-1.207484	-0.525148	32	728.82
33	8	4.251785	-1.349336	-0.861790	33	750.98
34	8	2.308555	-2.137561	-0.382617	34	768.73
					35	817.51
					36	827.66
					37	848.43
					38	862.63
					39	888.81
					40	905.29
					41	912.30
					42	919.01
					43	966.91
					44	1004.56
					45	1022.79
					46	1034.27
					47	1035.41
					48	1069.45
					49	1088.05
					50	1098.98
					51	1126.59
					52	1141.65
					53	1187.77
					54	1190.56
					55	1192.91
					56	1215.44
					57	1220.16
					58	1286.14
					59	1292.98
					60	1300.35
					61	1326.42
					62	1339.18
					63	1339.86
					64	1367.56
					65	1386.67
					66	1411.72
					67	1432.85

					68	1441.42
					69	1460.18
					70	1471.49
					71	1485.51
					72	1490.77
					73	1496.14
					74	1498.90
					75	1509.09
					76	1535.89
					77	1650.62
					78	1669.96
					79	1703.22
					80	1723.38
					81	1773.97
					82	3061.52
					83	3076.64
					84	3116.49
					85	3120.47
					86	3140.69
					87	3148.75
					88	3167.74
					89	3184.00
					90	3187.09
					91	3217.67
					92	3249.00
					93	3260.01
					94	3274.20
					95	3285.84
					96	3792.57

Sum of Electronic and Nuclear Energy: -934.849729 (Hartree)

Sum of Electronic and Thermal Free Energies: -934.625101

Standard Free Energy (1,0 M): -934.622082

Structure: 4a (Charge = -1)

Center Number	Atomic Symbol	Coordinates (Angstroms) X	Y	Z	Normal Mode	Frequencies (cm**-1)
1	6	2.439102	0.078999	-0.347339	1	14.41
2	6	3.021273	1.010722	-1.229658	2	35.33

3	6	2.787908	2.370692	-1.081810	3	41.03
4	6	1.960888	2.829819	-0.055917	4	58.43
5	6	1.345669	1.903540	0.786796	5	59.81
6	6	1.545620	0.528281	0.662034	6	75.61
7	1	1.779497	3.891206	0.078669	7	112.22
8	1	0.674541	2.262425	1.559731	8	126.84
9	6	0.702268	-0.432628	1.502617	9	150.62
10	6	-0.350456	-1.080041	0.607955	10	195.35
11	1	1.339845	-1.226038	1.884898	11	221.96
12	6	-0.268911	-2.345899	0.188894	12	231.49
13	1	-1.022088	-2.769549	-0.466784	13	232.40
14	1	0.577857	-2.957148	0.484912	14	262.93
15	8	0.112807	0.190869	2.632533	15	283.34
16	1	-0.606672	0.756469	2.311439	16	297.50
17	6	-1.490227	-0.208404	0.194501	17	340.55
18	8	-1.703421	0.889265	0.687890	18	358.51
19	8	-2.257755	-0.735062	-0.758180	19	362.25
20	6	-3.418800	0.019568	-1.162104	20	371.71
21	6	-4.572116	-0.175272	-0.192548	21	422.26
22	1	-3.664861	-0.371417	-2.151347	22	432.29
23	1	-3.144042	1.074342	-1.247379	23	495.93
24	6	-5.816985	0.566041	-0.677227	24	503.08
25	1	-4.779163	-1.247064	-0.101770	25	541.26
26	1	-4.274919	0.189500	0.795853	26	563.22
27	1	-6.646451	0.429157	0.020455	27	567.62
28	1	-5.625099	1.640059	-0.765203	28	603.40
29	1	-6.137306	0.198907	-1.657503	29	628.41
30	1	3.675501	0.634467	-2.006003	30	652.97
31	1	3.262673	3.072332	-1.761347	31	677.99
32	7	2.790400	-1.272336	-0.511238	32	705.53
33	8	3.296160	-1.653675	-1.636498	33	750.42
34	8	2.770522	-2.074374	0.502114	34	753.62
					35	763.75
					36	801.63
					37	831.63
					38	847.41
					39	861.77
					40	883.62
					41	903.91
					42	916.23
					43	963.76
					44	972.65

45	1003.74
46	1017.89
47	1040.32
48	1071.39
49	1076.81
50	1085.69
51	1109.04
52	1140.67
53	1152.83
54	1170.49
55	1191.18
56	1199.93
57	1213.60
58	1275.42
59	1292.87
60	1293.38
61	1296.80
62	1329.61
63	1335.34
64	1347.86
65	1366.71
66	1381.67
67	1411.32
68	1413.24
69	1417.28
70	1434.87
71	1456.88
72	1484.90
73	1494.19
74	1495.12
75	1497.94
76	1508.28
77	1527.03
78	1634.98
79	1665.72
80	1717.02
81	1774.58
82	3058.78
83	3073.83
84	3110.20
85	3116.76
86	3136.77

87	3146.63
88	3172.96
89	3186.39
90	3189.24
91	3204.36
92	3217.92
93	3230.37
94	3246.95
95	3288.47
96	3768.44

Sum of Electronic and Nuclear Energy: -934.975624 (Hartree)

Sum of Electronic and Thermal Free Energies: -934.755319

Standard Free Energy (1,0 M): -934.752300

Structure: 4b (Charge = 0)

Center Number	Atomic Symbol	Coordinates (Angstroms) X	Y	Z	Normal Mode	Frequencies (cm**-1)
1	6	1.766488	0.061162	0.503097	1	20.37
2	6	2.819056	-0.501198	-0.207507	2	26.66
3	6	3.376295	0.090635	-1.334638	3	42.84
4	6	2.843642	1.304673	-1.754750	4	47.30
5	6	1.785526	1.891735	-1.059928	5	68.12
6	6	1.237061	1.276181	0.068713	6	80.09
7	1	4.194920	-0.387184	-1.857878	7	93.87
8	1	3.255358	1.796240	-2.629381	8	109.84
9	1	1.379834	2.840293	-1.395059	9	159.59
10	6	0.105375	1.940861	0.852388	10	180.62
11	6	-0.904432	0.922183	1.345453	11	193.95
12	1	0.543954	2.423401	1.732617	12	211.84
13	6	-1.087654	0.639597	2.636350	13	248.94
14	1	-1.811535	-0.103114	2.954052	14	257.08
15	1	-0.511229	1.148780	3.403320	15	293.73
16	8	-0.509686	2.979586	0.116204	16	302.27
17	1	-0.979036	2.565162	-0.625626	17	343.18
18	6	-1.697821	0.250772	0.272843	18	354.99
19	8	-1.669095	0.626036	-0.888311	19	373.32
20	8	-2.423773	-0.776090	0.700310	20	418.13
21	6	-3.268744	-1.436939	-0.266740	21	428.15

22	6	-4.584136	-0.697932	-0.440836	22	454.69
23	1	-3.426615	-2.433850	0.148694	23	494.96
24	1	-2.726296	-1.518104	-1.212189	24	512.52
25	6	-5.498529	-1.445070	-1.410245	25	540.32
26	1	-5.065685	-0.600900	0.538049	26	558.61
27	1	-4.382886	0.312353	-0.812158	27	599.13
28	1	-6.450356	-0.922036	-1.527676	28	630.82
29	1	-5.036890	-1.531183	-2.398833	29	662.98
30	1	-5.712307	-2.455756	-1.048106	30	685.54
31	1	1.373602	-0.447425	1.376441	31	709.50
32	7	3.365408	-1.785293	0.253548	32	742.36
33	8	4.295695	-2.270099	-0.370358	33	757.06
34	8	2.862428	-2.305632	1.236369	34	766.99
					35	824.27
					36	838.98
					37	850.83
					38	863.80
					39	907.39
					40	912.07
					41	930.89
					42	949.02
					43	978.85
					44	981.81
					45	1018.66
					46	1025.97
					47	1032.99
					48	1036.37
					49	1070.21
					50	1118.92
					51	1126.37
					52	1137.84
					53	1142.84
					54	1186.52
					55	1194.60
					56	1224.75
					57	1233.66
					58	1275.28
					59	1293.79
					60	1307.32
					61	1319.81
					62	1330.87
					63	1340.85

64	1371.15
65	1390.26
66	1411.83
67	1419.39
68	1439.67
69	1455.85
70	1467.92
71	1485.82
72	1486.27
73	1498.15
74	1501.20
75	1510.64
76	1532.94
77	1654.51
78	1668.13
79	1714.91
80	1728.51
81	1784.22
82	3062.43
83	3078.36
84	3103.93
85	3114.45
86	3120.21
87	3140.87
88	3149.64
89	3179.93
90	3183.19
91	3221.53
92	3237.72
93	3246.43
94	3274.97
95	3279.20
96	3802.86

Sum of Electronic and Nuclear Energy:	-934.857550 (Hartree)
Sum of Electronic and Thermal Free Energies:	-934.634132
Standard Free Energy (1,0 M):	-934.631113

===

===

Structure: 4b (Charge = -1)

Center Atomic Coordinates (Angstroms) Normal Frequencies

Number	Symbol	X	Y	Z	Mode	(cm**-1)
1	6	1.743179	0.061397	0.493256	1	19.13
2	6	2.814439	-0.552902	-0.182928	2	30.37
3	6	3.387210	0.074990	-1.306620	3	48.37
4	6	2.883347	1.298321	-1.726195	4	67.73
5	6	1.819630	1.914848	-1.057865	5	80.03
6	6	1.248692	1.288223	0.055775	6	91.10
7	1	4.208082	-0.408214	-1.820837	7	99.09
8	1	3.325211	1.784907	-2.591161	8	113.43
9	1	1.439587	2.872507	-1.395518	9	162.45
10	6	0.117721	1.965560	0.829010	10	178.53
11	6	-0.903782	0.961644	1.327315	11	195.01
12	1	0.547832	2.458092	1.708227	12	213.83
13	6	-1.110188	0.705090	2.620110	13	252.60
14	1	-1.842777	-0.027020	2.942499	14	257.31
15	1	-0.541597	1.225974	3.385212	15	294.07
16	8	-0.501240	3.002004	0.084978	16	304.73
17	1	-0.927593	2.582654	-0.679025	17	344.04
18	6	-1.689872	0.275308	0.258961	18	351.94
19	8	-1.681762	0.647970	-0.903000	19	371.85
20	8	-2.394389	-0.766943	0.692637	20	373.50
21	6	-3.236388	-1.439305	-0.267223	21	420.90
22	6	-4.562001	-0.717188	-0.436990	22	444.76
23	1	-3.380926	-2.437230	0.150949	23	471.06
24	1	-2.699072	-1.517126	-1.216017	24	504.52
25	6	-5.473395	-1.479428	-1.397416	25	519.48
26	1	-5.038931	-0.621333	0.544348	26	548.44
27	1	-4.374863	0.293660	-0.813984	27	561.10
28	1	-6.431159	-0.967025	-1.513949	28	608.63
29	1	-5.015343	-1.567064	-2.387577	29	633.21
30	1	-5.675066	-2.490047	-1.028111	30	662.54
31	1	1.320316	-0.441627	1.354684	31	681.47
32	7	3.302253	-1.781464	0.257964	32	710.09
33	8	4.279779	-2.338134	-0.372416	33	720.81
34	8	2.764985	-2.346174	1.285220	34	757.20
					35	782.33
					36	815.15
					37	842.50
					38	851.88
					39	886.75
					40	901.94

41	908.48
42	918.51
43	933.76
44	978.75
45	1002.63
46	1003.63
47	1018.65
48	1037.89
49	1070.61
50	1102.98
51	1113.51
52	1118.86
53	1142.51
54	1181.83
55	1194.53
56	1217.63
57	1223.39
58	1271.79
59	1293.30
60	1297.39
61	1316.50
62	1330.55
63	1341.12
64	1351.25
65	1372.30
66	1388.22
67	1412.15
68	1418.46
69	1429.17
70	1439.67
71	1453.48
72	1485.88
73	1498.81
74	1499.32
75	1501.31
76	1510.66
77	1538.73
78	1645.36
79	1661.31
80	1729.84
81	1785.23
82	3062.90

83	3078.30
84	3097.18
85	3112.93
86	3120.24
87	3140.99
88	3150.37
89	3178.15
90	3182.13
91	3199.29
92	3241.35
93	3243.03
94	3269.74
95	3277.34
96	3803.29

Sum of Electronic and Nuclear Energy: -934.983299 (Hartree)

Sum of Electronic and Thermal Free Energies: -934.762349

Standard Free Energy (1,0 M): -934.759330

Structure: 4c (Charge = 0)

Center Number	Atomic Symbol	Coordinates (Angstroms) X	Y	Z	Normal Mode	Frequencies (cm**-1)
1	6	2.547947	-0.064612	1.185519	1	19.68
2	6	3.247693	-0.396418	0.030120	2	33.57
3	6	2.854133	0.035403	-1.230688	3	40.76
4	6	1.714818	0.826255	-1.332994	4	57.07
5	6	0.984163	1.173412	-0.193351	5	63.09
6	6	1.411923	0.724879	1.062104	6	77.22
7	6	-0.243012	2.075872	-0.303876	7	86.22
8	6	-1.379125	1.590660	0.576801	8	106.34
9	6	-1.818932	2.264412	1.640883	9	124.62
10	6	-1.989628	0.294700	0.154548	10	180.44
11	8	-0.659135	2.252657	-1.643321	11	199.24
12	8	-2.881474	-0.189473	1.011283	12	234.25
13	8	-1.683696	-0.256151	-0.890825	13	253.88
14	6	-3.557844	-1.411061	0.640505	14	267.20
15	6	-4.702031	-1.137735	-0.320357	15	288.70
16	1	3.428144	-0.241552	-2.106358	16	294.31
17	1	1.392830	1.180888	-2.305169	17	330.02

18	1	0.046758	3.071552	0.050058	18	346.36
19	1	-2.631010	1.886115	2.252254	19	364.55
20	1	-1.368644	3.212117	1.921976	20	416.42
21	1	-0.996216	1.396686	-1.952559	21	439.96
22	1	-3.923282	-1.814990	1.586286	22	477.60
23	1	-2.827773	-2.100738	0.208872	23	506.62
24	1	-4.303716	-0.689144	-1.236022	24	511.63
25	1	-5.380679	-0.410071	0.137415	25	523.29
26	1	0.852854	0.991697	1.953653	26	548.38
27	1	2.887960	-0.419218	2.150660	27	604.50
28	8	5.057905	-1.509130	-0.872125	28	628.11
29	8	4.780221	-1.605746	1.261416	29	638.57
30	7	4.447792	-1.230088	0.148115	30	678.66
31	6	-5.451735	-2.428315	-0.647899	31	713.47
32	1	-4.788049	-3.159051	-1.120682	32	738.09
33	1	-5.865599	-2.883253	0.257561	33	757.04
34	1	-6.278904	-2.232390	-1.334059	34	766.57
---					35	826.11
					36	844.82
					37	860.32
					38	874.01
					39	887.60
					40	907.55
					41	910.52
					42	922.43
					43	965.62
					44	1013.25
					45	1021.29
					46	1027.60
					47	1033.28
					48	1034.74
					49	1070.59
					50	1113.89
					51	1139.63
					52	1143.40
					53	1148.48
					54	1194.87
					55	1204.01
					56	1220.75
					57	1225.56
					58	1273.97
					59	1295.07

60	1305.12
61	1324.00
62	1332.40
63	1341.17
64	1372.37
65	1390.18
66	1412.91
67	1415.72
68	1438.25
69	1451.68
70	1461.70
71	1465.13
72	1487.20
73	1494.05
74	1502.18
75	1511.74
76	1542.75
77	1645.57
78	1682.65
79	1703.81
80	1729.35
81	1780.28
82	3061.50
83	3079.59
84	3099.75
85	3116.99
86	3122.02
87	3141.44
88	3149.18
89	3176.19
90	3181.89
91	3231.87
92	3244.94
93	3266.53
94	3274.29
95	3274.56
96	3798.28

-- -----------------

Sum of Electronic and Nuclear Energy: -934.857843 (Hartree)

Sum of Electronic and Thermal Free Energies: -934.634644

Standard Free Energy (1,0 M): -934.631625

===

==

Structure: 4c (Charge = -1)

Center Number	Atomic Symbol	X	Y	Z	Normal Mode	Frequencies (cm**-1)
1	6	2.534644	-0.105831	1.185566	1	16.11
2	6	3.270169	-0.422589	0.025950	2	30.58
3	6	2.851603	0.089670	-1.216767	3	41.83
4	6	1.721768	0.896593	-1.288141	4	64.59
5	6	0.979697	1.213022	-0.144947	5	72.61
6	6	1.409339	0.700104	1.087475	6	83.89
7	6	-0.240585	2.125585	-0.214992	7	96.03
8	6	-1.389000	1.576563	0.611668	8	109.45
9	6	-1.856536	2.178095	1.707171	9	121.58
10	6	-1.985476	0.303620	0.106307	10	174.79
11	8	-0.647184	2.389514	-1.547523	11	194.57
12	8	-2.793497	-0.296545	0.977047	12	249.16
13	8	-1.751482	-0.135015	-1.007966	13	250.37
14	6	-3.470929	-1.491268	0.534138	14	260.59
15	6	-4.716009	-1.150803	-0.266838	15	281.22
16	1	3.421586	-0.155209	-2.104162	16	291.95
17	1	1.412409	1.290850	-2.251034	17	330.50
18	1	0.023370	3.102876	0.205616	18	345.27
19	1	-2.670908	1.752341	2.283394	19	359.59
20	1	-1.427186	3.115549	2.049232	20	381.04
21	1	-0.953850	1.546839	-1.918225	21	426.49
22	1	-3.726044	-2.016800	1.456412	22	458.03
23	1	-2.773636	-2.098670	-0.048688	23	497.36
24	1	-4.426588	-0.584834	-1.158313	24	503.56
25	1	-5.359089	-0.505063	0.340636	25	525.76
26	1	0.851321	0.934337	1.990978	26	534.63
27	1	2.861524	-0.500769	2.139133	27	549.19
28	8	5.068257	-1.506321	-0.957504	28	607.21
29	8	4.760193	-1.698114	1.259655	29	629.46
30	7	4.399825	-1.232972	0.111349	30	640.55
31	6	-5.466279	-2.420145	-0.667203	31	673.33
32	1	-4.839723	-3.066729	-1.289453	32	712.96
33	1	-5.770404	-2.991998	0.215202	33	719.17
34	1	-6.366287	-2.175766	-1.236149	34	752.45
					35	794.46
					36	826.33

37	840.68
38	850.54
39	855.33
40	881.23
41	909.57
42	918.23
43	961.88
44	985.71
45	1003.61
46	1018.92
47	1019.30
48	1036.08
49	1071.83
50	1097.12
51	1104.81
52	1125.86
53	1143.82
54	1188.31
55	1192.72
56	1218.21
57	1223.73
58	1271.26
59	1292.78
60	1298.62
61	1306.95
62	1328.99
63	1332.58
64	1337.32
65	1373.41
66	1384.30
67	1417.24
68	1417.78
69	1435.13
70	1441.96
71	1451.54
72	1463.47
73	1488.98
74	1499.06
75	1500.98
76	1511.67
77	1549.85
78	1632.12

```
                                                     79      1665.94
                                                     80      1724.13
                                                     81      1781.80
                                                     82      3071.29
                                                     83      3083.35
                                                     84      3097.01
                                                     85      3109.09
                                                     86      3125.30
                                                     87      3148.00
                                                     88      3156.92
                                                     89      3170.78
                                                     90      3181.42
                                                     91      3202.10
                                                     92      3218.96
                                                     93      3258.33
                                                     94      3264.54
                                                     95      3277.28
                                                     96      3797.49
------------------------------------------------- ------------------
Sum of Electronic and Nuclear Energy:            -934.982483 (Hartree)
Sum of Electronic and Thermal Free Energies:     -934.762636
Standard Free Energy (1,0 M):                    -934.759617
====================================================================
```
